# Preparation and photophysical properties of quinazoline-based fluorophores†

**DOI:** 10.1039/d0ra05701k

**Published:** 2020-08-17

**Authors:** Zhichao Wang, Hanjie Li, Zhixing Peng, Zaibin Wang, Yanguang Wang, Ping Lu

**Affiliations:** Department of Chemistry, Zhejiang University Hangzhou 310027 P. R. China pinglu@zju.edu.cn orgwyg@zju.edu.cn

## Abstract

The donor–acceptor design is a classic method of synthesizing new fluorescent molecules. In this study, a series of new fluorescent compounds (1–10) were synthesized based on 2-(3,5-bis(trifluoromethyl)phenyl)-quinazoline acceptor and various amino donors. The fluorescent emissions of 1–10 cover the spectrum from 414 nm to 597 nm in cyclohexane solutions with various amino donors on 4- or 7-positions of quinazoline. Ultimately, compounds 1 and 2 presented the highest photoluminescence quantum yield (QY) over 80%, while compound 10 provided the largest Stokes shift (161 nm) in cyclohexane. Most of them have strong emissions in aggregated states such as in nanoparticles, in powders, in crystals and in films. Mechanochromic properties were observed for compounds 1, 2, 4 and 7. Furthermore, blue OLEDs were fabricated by using compound 2 or 7 as the active layer.

## Abstract 2

A series of new fluorescent compounds were synthesized based on 2-(3,5-bis(trifluoromethyl)phenyl)-quinazoline, with alterable emission, high quantum yield, mechanochromic properties and can be applied as an optoelectronic material for blue OLED.

## Graphical abstract

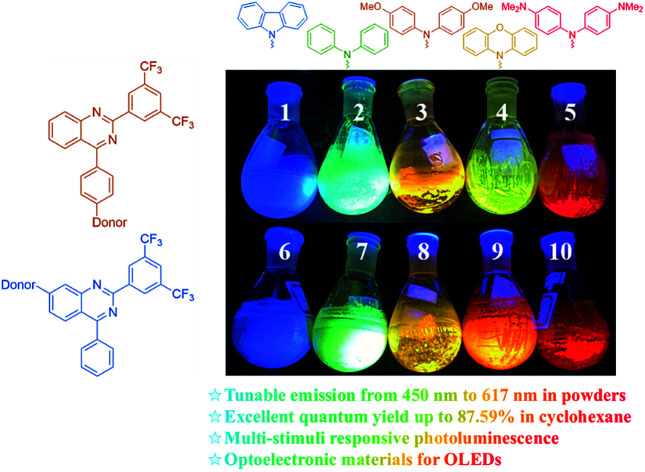

## Introduction

Quinazoline is one of the most attractive heterocycles in alkaloids. Their derivatives are abundant in natural products and show a broad spectrum of bioactivities in living organisms.^1–5^ For instance, some quinazoline-based compounds exhibited effective and selective CYP1A2 inhibition and might function in cancer chemoprevention.^6^ CID 9998128 (2,4-disubstituted quinazoline) has been revealed as a good candidate for Alzheimer's disease (AD) treatment.^7^ Besides the unique attributes of quinazolines in medicinal chemistry, a series of fluorescent quinazolines have also been designed and synthesized for optoelectronic devices^8–10^ and fluorescent chemosensors.^11–14^ Recently, quinazoline was successfully functioned as an anchor to prevent the stacking between fluorophores and turned aggregation-caused quenching (ACQ) to aggregation-enhanced emission (AEE).^15^ Covalently bonded with coumarin, the coumarin–quinazoline dyad displayed solvatochromic and ACQ properties. Taking advantage of this disaggregation-induced emission (DIE), a turn-on fluorescent probe was designed and was capable of probing parallel G4 DNA topologies over other G4 DNA structures.^16^ However, in contrast to frequently used electron-deficient benzothiadiazole (BTD),^17^ the fluorescent compounds based on quinazolines largely remained immature. The same goes for the fluorescent structure–property relationship (FSPR)^18,19^ studies which are instructive in the development of new fluorophores with a unique application.

The multicomponent reaction provided a new avenue in the construction of quinazolines with various substituents in a single step and made the investigation and establishment of FSPR practical and possible.^20^ For instance, assisted by CuCl_2_, 2,4-diarylquinazolines could be effectively prepared from 2-methylquinoline, 2-aminobenzophenome, ammonia acetate and oxygen in a single step.^21^ 2,3-Dihydroquinazolin-4(1*H*)-ones could be efficiently prepared by three component reaction from isatoic anhydride, aniline and arylaldehyde in the presence of Al_2_(SO_4_)_3_ with a variety of functional groups.^22^ In the presence of Cu(OTf)_2_, a [2 + 2 + 2] cascade annulation of diaryliodonium salts with two nitriles produced 2,4-disubstituted quinazolines in excellent yields.^23^ Mediated by 4-hydroxy-TEMPO radical^24^ or iodine,^25^ 2-aryl-quinazolines could be obtained from arylmethanamines, 2-aminobenzoketones and oxidant. Here, we report the preparation procedure for 2,4-diarylquinazolines and 2,4,7-triarylquinazolines and their fluorescent properties as well as their applications in OLED.

## Results and discussion

### Synthesis of compounds 1–10

**Scheme 1:**
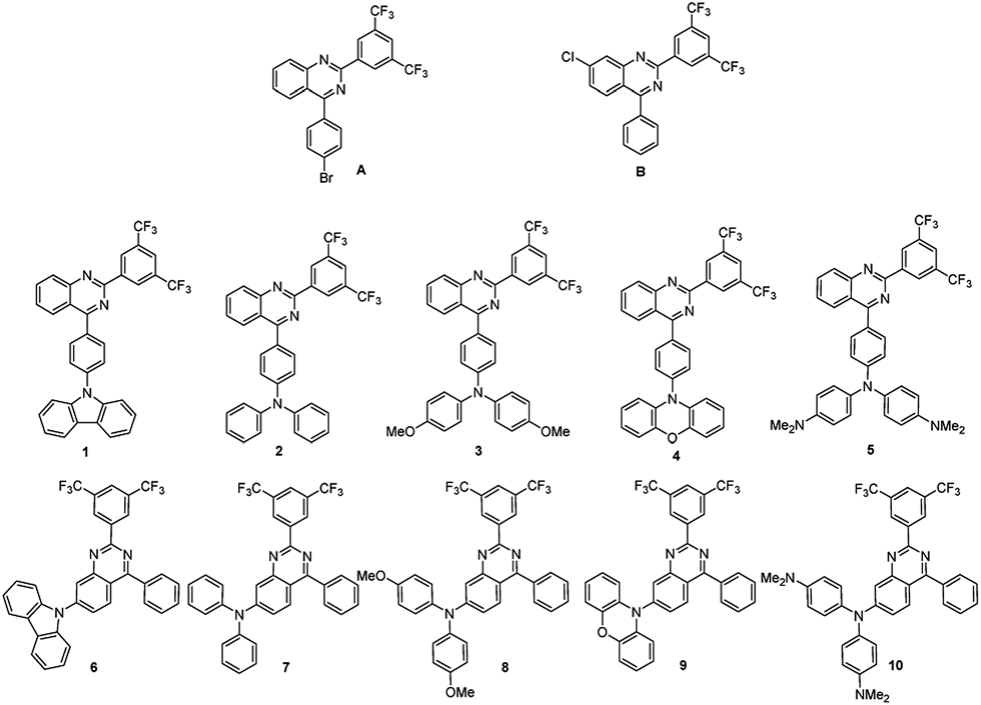
Key intermediates A, B and compounds 1–10.

The preparatory routes leading to compounds 1–10 were shown in Fig. S1† and structures of intermediates A and B as well as the structures of compounds 1–10 were presented in Scheme 1. Key intermediates A and B were synthesized according to the literature reporting palladium-catalyzed three components reaction starting from arylboronic acids, cyanoanilines and arylaldehydes.^26^ In this way, A and B were prepared in yields of 60% and 70%, respectively. Subsequent Buchwald–Hartwig coupling of A or B with various secondary aromatic amines afforded target molecules 1–10 in yields varying from 84% to 99%. Structures of these synthesized compounds (A, B, 1–10) were confirmed by ^1^H NMR, ^13^C NMR and ^19^F NMR (Fig. S2–S37†). Further confirmation was made by HRMS and IR as well as single crystal analysis of compounds 1–4 (Table S1†). These compounds are thermally stable (Fig. S38† and Table 1). The decomposition temperatures were determined to be 369 °C, 380 °C, 382 °C, 363 °C, 383 °C, 377 °C, 342 °C, 379 °C, 351 °C and 393 °C for compounds 1–10, respectively.

**Table 1:** Photophysical property of 1–10 in cyclohexanes and thermal stability of 1–10

Compounds	Absorption^a^		Emission						*T*_d_ f (°C)
	*λ*_max_ (nm)	*ε* (L mol^−1^ cm^−1^)	*λ*_max_ a ^,^ b (nm)	Stokes (nm cm^−1^)	Life time^c^ (ns)	QY^d^ (%)	*k*_r_ e (10^7^ s^−1^)	*k*_nr_ e (10^7^ s^−1^)	
1	338, 369	9200, 9550	414	45/2982	3.37	84.67	25.1	4.5	369
2	403	18 050	450	47/2592	3.96	87.59	22.1	3.1	380
3	415	13 900	500	85/4079	6.03	22.75	3.8	12.8	382
4	420	1150	514	94/4354	1.32(40.4%)	21.94	2.9	10.2	363
					9.71(59.6%)				
5	444	7500	575	131/5152	4.1	7.35	1.8	22.6	383
6	378, 397	5200, 5550	412	15/930	3.29	18.80	5.7	24.5	377
7	400	6550	458	58/3153	5.81	43.32	7.5	9.8	342
8	411	6100	519	108/5063	8.36	19.30	2.3	9.7	379
9	456	750	533	77/3168	1.18(52.9%)	16.26	2.7	14	351
					9.06(47.1%)				
10	436	4550	597	161/6185	4.31	17.57	4.1	19.1	393

a Measured in cyclohexane at 2 × 10^−5^ M.

b Excited at 320 nm (4 and 9), 445 nm (5) and 365 nm (1–3, 6–8, and 10).

c According to fluorescence decay traces of compounds in cyclohexane (5, 10) and toluene (1–4 and 6–9) at 2 × 10^−5^ M.

d Quantum yields were obtained from an integrating sphere.

e Calculated by QY = *τk*_r_ = *k*_r_/(*k*_r_ + *k*_nr_).

f *T*_d_ was determined at 5% weight loss.

### Absorption and emission of 1–10 in cyclohexanes

**Fig. 1:**
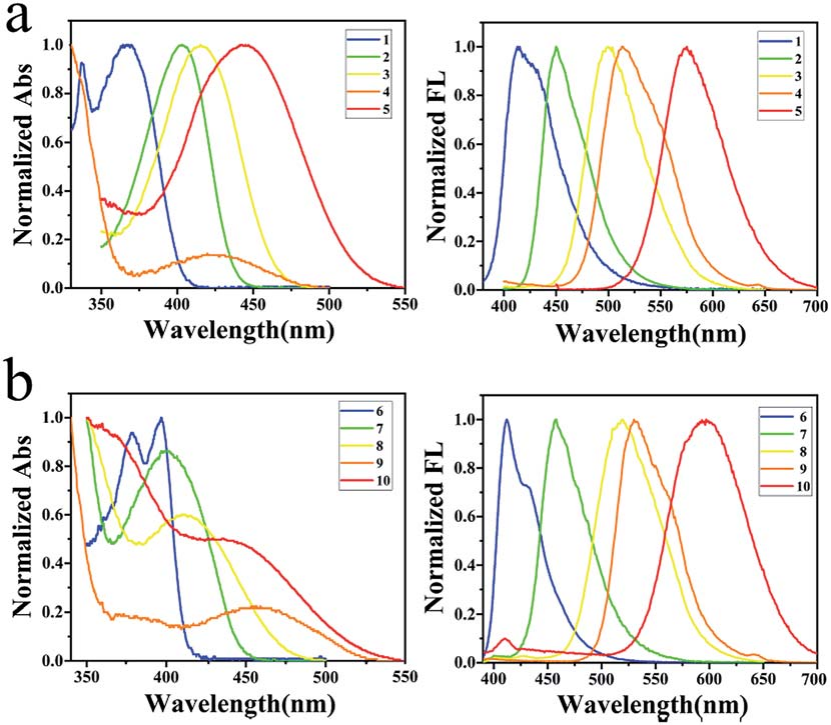
(a) Absorptions and normalized emissions of 1–5 in cyclohexanes; (b) absorptions and normalized emissions of 6–10 in cyclohexanes.

All the absorption and emission spectra were taken in a concentration of 2 × 10^−5^ M in order to avoid the intermolecular interactions between solutes (Fig. S39–S40 and Table S2†). Compound 1 absorbs lights at 338 nm and 369 nm with molar absorptivities of 9200 and 9550 L mol^−1^ cm^−1^ in cyclohexane (Table 1). Red-shifted absorption was observed when carbazole (CZ) was changed into diphenylamino (PA), di(4-methoxyphenyl)amino (MPA), phenoxazinyl (POZ) and di(4-(*N*,*N*-dimethylamino)phenyl)amino (DMPA). Thus, compounds 2, 3, 4 and 5 absorb lights in dilute cyclohexanes at 403, 415, 420 and 444 nm with molar absorptivities of 18 050, 13 900, 1150 and 7500 L mol^−1^ cm^−1^, respectively (Fig. 1a). Similar results were found for the series of compounds 6–10 when these amino groups occupied on the 7-position of quinazoline (Fig. 1b). It indicates that DMPA is the strongest electron-donating group among these secondary aromatic amines.

Compounds 1–10 are highly fluorescent in dilute solutions. Compound 1 emits light at 414 nm with Stokes shift of 45 nm (2982 cm^−1^) and quantum yield of 84.67%. As the amino group changed to better electron-donating groups, compounds 2, 3, 4 and 5 emit lights at 450, 500, 514 and 575 nm with Stokes shifts of 47 (2592), 85 (4079), 94 (4354) and 131 nm (5152 cm^−1^), respectively (Fig. 1a). The highest emission quantum yield (87.59%) was found for compound 2 with MPA occupied on the 4-position of quinazoline. Similar results were observed for compounds 6–10. These compounds emit lights at 412, 458, 519, 533 and 597 nm with Stokes shifts of 15 (930), 58 (3153), 108 (5063), 77 (3168) and 161 nm (6185 cm^−1^), respectively (Fig. 1b). Among this series, compound 7 presented the highest emission quantum yield (43.32%). To both series, emission spectra fit the single exponential function with fluorescence lifetime varying from 3.29 to 8.36 ns except for compounds 4 and 9 with POZ substituted (Fig. S41†). The decay trace is bi-exponential with lifetimes of 1.32 ns (40.4%) and 9.71 ns (59.6%) for the emission band at 514 nm of compound 4. The average lifetime was calculated to be 7.62 ns. The average life is calculated using the literature method.^27^

For compound 9, lifetimes of 1.18 ns (52.9%) and 9.06 ns (47.1%) were recorded and the average lifetime was calculated to be 5.98 ns. That both 4 and 9 exhibited emissions with bi-components might be the respective decays from S_1_ and T_1_ due to the phenoxazine-caused steric repulsion between hydrogens at donor–acceptor linkage and the resulted large dihedral angle between donor and acceptor.^28^ Tuning the donor on the 4-position of quinazoline affected the absorption spectra to a greater extent, while the larger Stokes shifts were observed for those compounds with the donor on 7-position except for compounds 6 and 9. Finally, compound 10 provided the largest Stokes shift with a value of 161 nm in cyclohexane.

### Absorption and emission of 1–10 in various solvents

**Fig. 2:**
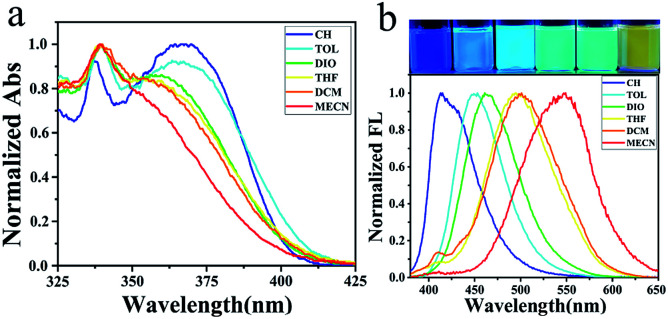
(a) Absorptions and (b) rainbow emissions of 1 in various solvents.

These compounds are soluble in normal organic solvents, such as cyclohexane (CH), toluene (TOL), dioxane (DIO), tetrahydro-furan (THF), dichloromethane (DCM) and acetonitrile (MeCN). Blue-shifted absorption and red-shifted emission of these D–A compounds were observed as the solvent polarity increased from cyclohexane to toluene, dioxane, tetrahydrofuran, dichloromethane and acetonitrile (Fig. S39, S40 and Table S2†). Meanwhile, rainbow emissions were recorded and could be clearly photographed under UV light as the solvent polarity changes. As a typical example, compound 1 absorbs light at 338/369, 340/363, 339/358, 339/354, 340/354 and 339 nm (Fig. 2a), while emits light at 414, 453, 461, 495, 501 and 548 nm in CH, TOL, DIO, THF, DCM and MeCN, respectively (Fig. 2b). The absorption at the lower energy gap of 1 disappeared as the solvent changed to MeCN. Finally, the largest Stokes shift (209 nm) was found for 1 in MeCN.^29^ As the solvent polarity increases, the HOMO/LUMO levels of these D–A compounds decrease through the salvation of compounds both in ground states and in excited states. However, in a more polar solvent, a larger energy gap was found for D–A compounds in ground state because of the more stabilization of the HOMO level, while a smaller energy gap for D–A compounds in excited states was observed because of the more stabilization of the LUMO level. Thus, blue-shifted absorption and red-shifted emission were presented for these D–A compounds as the solvent polarity increases.

### Emission of 1–10 in aggregates

**Fig. 3:**
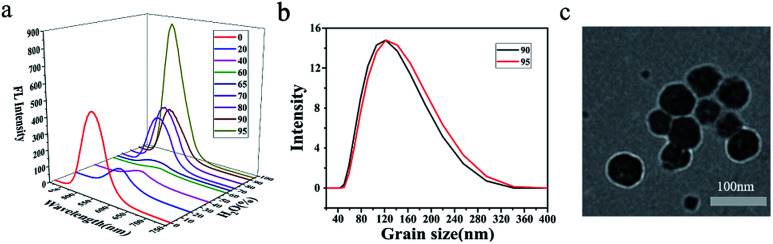
(a) 3D emissions of 2, (b) DLS (*f*_w_ = 90%, 95%) of 2 and (c) TEM images of 2 (95%).

Nanoparticles were prepared by quickly pouring water into their dilute THF solutions and their emission spectra were recorded accordingly (Fig. S42 and S43†). The shape and size were photographed by TEM (Fig. S44†) and recorded by DLS as well. Emissions of aggregates could be classified into two categories in general. For those compounds with strong fluorescence in dilute solutions, such as compounds 1, 2, 6 and 7, aggregation-causing quenching (ACQ) was firstly observed and followed by aggregation-induced emission enhancement (AIEE) as the water fraction increases. As the water fraction increase to 60%, the emission of compound 2 red-shifted to 564 nm and the emission intensity decreased to 5.1% of its original value (Fig. 3a). ACQ was observed in this stage. Further increment of water fraction to 95%, a steady blue-shift emission as well as the increment of the emission intensity was observed. Nanoparticles formed in ball shape with the diameter about 100 nm, determined by DLS (Fig. 3b) and confirmed by TEM (Fig. 3c). A similar situation was observed for compounds 1, 6 and 7. To those compounds with weak or non-fluorescent in dilute THF solutions (3–5, 8–10), increment of water fraction induced emission enhancement significantly. Compound 3 presented the typical aggregation-induced emission (AIE).^30^ As the water fraction increased to 60%, the fluorescence of compound 3 was turned on. Beyond this point, a steady increment of emission intensity was recorded. As the water fraction reached 90%, the emission intensity was magnified 21.6 times of its initial.

### Emission of 1–10 in powders

**Fig. 4:**
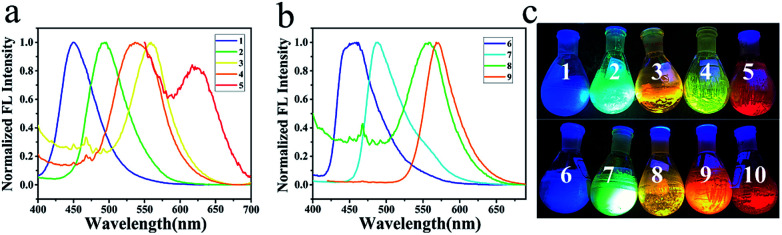
(a) Powder emission spectra of 1–5; (b) powder emission spectra of 6–9; (c) colorful powders of 1–10 under UV light.

These compounds are also strongly fluorescent in powders (Fig. 4 and Table 2). As the electron-donating group on 4-position of quinazoline changes from CZ to PA, MPA, and POZ, the maximum emission wavelengths in powders were determined to be 450, 495, 558 and 539 nm, respectively (Fig. 4a). When DMPA occupied on the 4-position of quinazoline, the maximum emission wavelength of 5 in powder was determined to be 467 nm with a shoulder peak (620 nm) at a larger energy gap. A colourful solid emission was photographed under UV light, altering the colour from bright blue to green, orange, yellow and finally to red, as the secondary aromatic amine changed on the 4-position of quinazoline (Fig. 4c). Emission quantum yields were determined to be 56.55%, 49.91%, 28.03%, 27.51% and 3.08% with the lifetimes of 4.34, 2.86, 5.44, 15.04 and 2.45 ns for compounds 1–5, respectively. For the series of electron-donating compounds on the 7-position of quinazoline, compounds 6–9 exhibited similar emissions with the colour changed from bright blue to red, but with relatively lower quantum yields, accordingly (Fig. 4b and c). As for compound 10, the emission presented weak and cannot be detected.

**Table 2:** Absorption in film and emission in film, powder and crystalline

Compounds	Emission in powder					Absorption in film *λ*_max_^e^ (nm)	Emission in film					Emission in crystalline *λ*_max_^f^ (nm)
	*λ*_max_ a (nm)	Life time^b^ (ns)	QY^c^ (%)	*k*_r_ d (10^7^ s^−1^)	*k*_nr_ d (10^7^ s^−1^)		*λ*_max_ f (nm)	Life time^b^ (ns)	QY^c^ (%)	*k*_r_ d (10^7^ s^−1^)	*k*_nr_ d (10^7^ s^−1^)	
1	450	4.34	56.55	13.0	10.0	364	467	5.04	31.55	6.3	13.6	468
2	495	2.86	49.91	17.5	17.5	410	509	3.00	26.06	8.7	24.6	491
3	559	5.44	28.03	5.2	13.2	429	555	4.84	25.99	5.4	15.3	539
4	540	15.04	27.51	1.8	4.8	412	506	0.48(60.8%)	20.86	8.0	30.2	532
								3.94(39.2%)				
5	617	2.45	3.08	1.3	39.6	413	—g	—g	—g	— g	—g	—h
6	460	2.34	15.21	6.5	36.2	388	467	1.31	32.84	25.1	51.3	—h
7	488	4.88	31.70	6.5	14.0	416	536	3.34	35.70	10.7	19.3	—h
8	558	7.61	17.92	2.4	10.8	430	543	7.4	11.35	1.5	12.0	—h
9	568	9.58	11.33	1.2	9.3	455	540	0.65(6.5%)	17.96	4.0	18.2	—h
								5.28(93.5%)				
10	—g	—g	—g	—g	—g	—g	—g	—g	—g	—g	—g	—h

a Excited at 380 nm (9), 400 nm (5) and 365 nm (1–4, 6–8 and 10).

b According to fluorescence decay traces.

c Quantum yields were obtained from an integrating sphere.

d Calculated by QY = *τk*_r_ = *k*_r_/(*k*_r_ + *k*_nr_).

e Films were prepared by spin-coating a dilute DCM solution on a quartz plate.

f Excited at 365 nm.

g Emission is too weak to be detected.

h No single crystal was obtained.

### Emission of 1–10 in film

The transparent films of these compounds were fabricated by dipping their dilute methylene chloride solutions onto quartz plates and let it evaporate to dry afterwards. For the series of amines occupied on the 4-position of quinazoline, emissions of compounds 1–5 in films were red-shifted in contrast to emissions of these compounds in powders, while quantum yields decreased accordingly too (Table 2). Film emission of compound 4 was bi-exponential with the combination of 0.48 ns (60.8%) and 3.94 ns (39.2%). The average lifetime was calculated to be 2.62 ns. Film of compound 5 presented weak fluorescence and the quantum yield was not detectable. For the series of the substituent on 7-position of quinazoline, films of compounds 6 and 7 exhibited larger quantum yields with respect to those in powders. Film emission of compound 9 also showed bi-exponential with the combination of 0.65 ns (6.5%) and 5.28 ns (93.5%). The average lifetime was calculated to be 4.50 ns.

### Emission of 1–4 in crystalline

Four single crystals (1–4) were obtained by slow evaporation of the dilute solutions of dichloromethane and hexane. The single crystal data were shown in Table S1.† Four single crystals are all granular, of which compound 1, 3 and 4 are clusters of small particles, and compound 2 are large regular crystals.

**Fig. 5:**
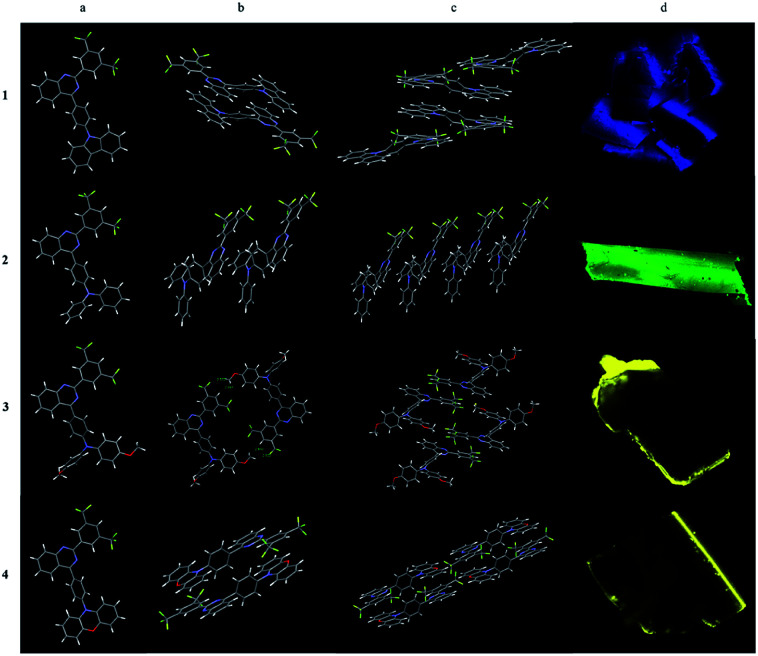
Single crystal structures of 1–4: (a) monomeric structure; (b) dimeric structures; (c) tetrameric structures and (d) CLSM images under 405 nm laser of 1–4.

As shown in Fig. 5, the dimers of 1, 4 are stacked together in an anti-parallel manner that might be the nature of the planarity of carbazole in 1 and phenoxazine in 4. Intermolecular π–π stacking between carbazole and quinazoline in molecule 1 was observed at a distance of 3.690 Å. A shorter distance (3.545 Å) in 4 was measured between phenoxazine and quinazoline which might be the dislocated alignment arising from the larger dihedral angle (72.71°) between phenylene and phenoxazine in comparison with that in 1 between phenylene and carbazole (40.84°). Finally, multiple C–H⋯π and C–H⋯F interactions hold molecules together. The stacking of molecule 4 was additionally assisted by the methylene chloride solvent in which C–H⋯Cl interactions were seen. Single crystal 1 emits light at 468 nm which is similar to its film emission, while single crystal 4 emits light at 532 nm which is red-shifted in comparison with its film emission in 506 nm.

Because of the propeller structure of triarylamine, intermolecular π–π stacking between donor and acceptor were not seen in the cases of 2 and 3. The main interactions are intermolecular C–H⋯π and C–H⋯F interactions. Thus, both of them showed blue-shifted emissions in comparison to their film emissions. Single crystal 2 emits light at 491 nm while single crystal 3 emits light at 539 nm (Table 2).

### Mechanochromic properties of 2 and 4

**Fig. 6:**
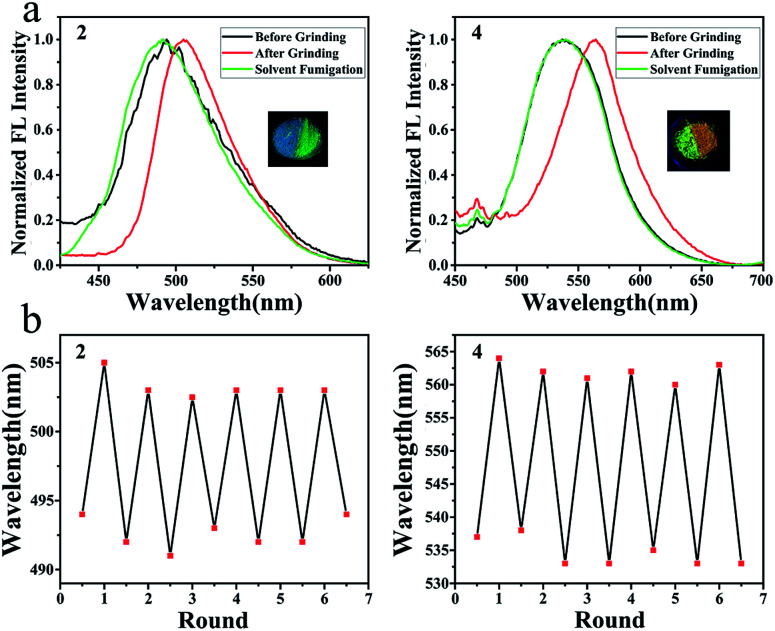
Mechanochromic property of compounds 2 and 4; (a) normalized emission spectra; (b) emission cycles.

Some of them showed mechanochromic property, such as compounds 1, 2, 4 and 7 (Fig. 6 and S45†). While grinding the crystalline of compound 2, the maximum emission wavelength changed from 494 nm to 505 nm and emission colour under UV light changed from blue to green (Fig. 6a). After methylene chloride fumigation, maximum emission wavelength changed back to 492 nm while the colour was turning back to blue (Fig. 6a). The cycle of blue and green was reproducible without the loss of the emission intensity (Fig. 6b). SAXS analysis indicated that the phase alternation existed during the grinding and fumigation process (Fig. S46†). Grinding crystalline of compound 4 altered the maximum emission wavelength from 533 nm to 564 nm with the colour alternation from yellow to brown (Fig. 6a). The colour could be tuned back to yellow by fuming the sample with DCM vapour (Fig. 6b).

### Analysis of HOMO/LUMO orbitals of 1–10

**Fig. 7:**
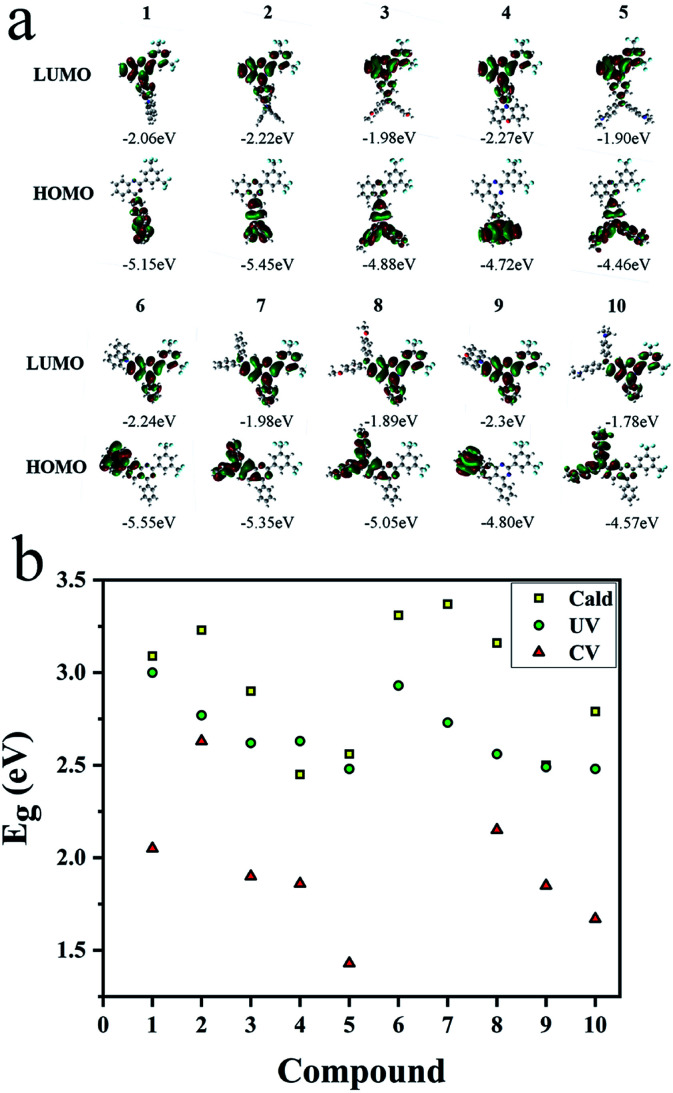
(a) HOMO/LUMO orbitals calculated by Gaussian (DFT using the B3LYP functional and the 6-31G(d) basis set). (b) Energy gaps (HOMO/LUMO) from theoretical calculation, UV observation and CV *versus* compounds 1–10.

Three methods were used to evaluate the HOMO/LUMO orbitals, including UV (Fig. S39 and S40†) and CV measurements (Fig. S47†) as well as the theoretical calculation (Fig. 7a) for comparison. Results were listed in Table S3.† Based on the calculation, donor and acceptor are clearly separated by HOMO and LUMO orbital distributions. Energy gaps obtained from CV and UV measurements are in good accordance with the results from calculation (Fig. 7b). Gradually decreased energy gaps from 1 to 5 as well as from 6 to 10 explained the full spectrum emissions of these compounds in solutions (Fig. 1) and in solids (Fig. 4c).

### OLED performance

Considering the quantum yields of solids, the flatness of the films and the HOMO/LUMO levels, we chose a pair of compounds 2 and 7 for the OLED fabrication (Fig. S48†). The devices were structured of ITO/PEDOT: PSS (40 nm)/compound (65 nm)/TBPI (40 nm)/LiF (1.5 nm)/Al (50 nm) and the device performances were listed in Table S4.† When the pure compound 2 was sandwiched in a thickness of 65 nm, device A emitted light at 509 nm with the EQE of 0.47% and the turn-on voltage of 4.5 V. By doping compound 2 in PVK (2 : PVK = 1 : 4) as emitter, device B emitted light at 513 nm. Increased EQE (1.09%) and decreased turn-on voltage (4.2 V) were recorded. As the ratio of 2 : PVK changed from 1 : 4 to 1 : 8 (device C), the EQE value was further increased to 1.35%. Device D, fabricated with the emitter of 7 in PVK (7 : PVK = 1 : 4) emitted light at 510 nm with the EQE of 0.83% and the turn-on voltage of 4.8 V.

## Conclusions

In this work, 4- or 7-donor substituted quinazolines were synthesized for the investigation of the fluorescent structure–property relationship of quinazoline-based donor–acceptor compounds. These compounds are thermally stable and could be dissolved in normal organic solvents which showed the potential applications in devices for the solution-processible. Moreover, these quinazolines-based D–A compounds emit bright light in different states with excellent quantum yields. The emission colour could be finely tuned by the intrinsic substituent, the extrinsic solvent polarity and grinding. Moreover, some of them might be used as the emissive material in the fabrication of OLEDs.

## Conflicts of interest

The authors declare no conflict of interest.

## Supplementary Material

RA-010-D0RA05701K-s001

RA-010-D0RA05701K-s002
