# Supplementary material for: Preparation and photophysical properties of quinazoline-based fluorophores
Source: RSC Adv. 2020 Aug 17;10(51):30297–303. doi: 10.1039/d0ra05701k (PMC9056281; doi:10.1039/d0ra05701k)
Supplement: RA-010-D0RA05701K-s001 [file RA-010-D0RA05701K-s001.pdf]

Electronic Supplementary Material (ESI) for RSC Advances.
This journal is © The Royal Society of Chemistry 2020

Supporting information

Preparation and photophysical properties of quinazoline-based fluorophores

Zhichao Wang, Hanjie Li, Zhixing Peng, Zaibin Wang, Yanguang Wang*, Ping Lu*

Department of Chemistry, Zhejiang University, Hangzhou 310027, P. R. China

Content

1. **Experimental Procedures**
2. **Figure S1** Synthesis of quinazolines **A, B** and compounds **1-10**.
3. **Figures S2-37** ^{1}H NMR, ^{13}C NMR and ^{19}F NMR of quinazolines **A, B** and compounds **1-10**.
4. **Table S1** Single crystal data of compounds **1-4**.
5. **Figure S38** Thermogravimetric Analysis of ten compounds.
6. **Figures S39-40** Photophysical properties of compounds **1-10** in different solvent.
7. **Table S2** Solvent effect on absorption and emission of ten compounds.
8. **Figure S41** Fluorescence decay traces of ten compounds in cyclohexane (compounds 5 and 10) or toluene (other compounds) at 2 x 10^{-5} M.
9. **Figures S42-43** (a) 3D Emission spectrum in aqueous THF solutions, (b) the relative intensity and photos and (c) DLS (fw = 90%, 95%) of compounds **1-5** and **6-10**.
10. **Figure S44** TEM image in aqueous THF solutions of **2** (fw = 95%), **3** (fw = 95%), **4** (fw = 90%), **6** (fw = 80%), **7** (fw = 80%), **9** (fw = 95%).
11. **Figure S45** Mechanochromic property of compounds **1, 2, 4** and **7**; a) normalized emission spectra; b) emission cycles.
12. **Figure S46** SAXS analysis in the cycle of pristine (a) and ground (b), repeatable cycles, monitored by emission spectra (c) emission cycles.
13. **Figure S47** Cyclic voltammograms of ten compounds.
14. **Table S3** HOMO/LUMO levels calculated from theoretical calculation and CV as well as UV measurements.
15. **Figure S48** C-I-V curves of devices **A, B, C** and **D**.
16. **Table S4** Device performance.

Experimental Procedures

Material and Instruments

Unless otherwise mentioned, solvents and reagents were purchased from commercial sources and used as received. For the purpose of the measurement of photophysical properties, THF was distilled from sodium. Melting points were measured with a micro melting point apparatus. Infrared spectra were obtained with an FTIR spectrometer. NMR spectra were operated at 400 MHz for ^{1}H NMR, 101 MHz for ^{13}C NMR, 376 MHz for ^{19}F NMR. All the NMR spectra were recorded at room temperature. Chemical shifts were quoted in parts per million (ppm) referred to the appropriate solvent peak or 0.0 ppm for tetramethylsilane. The following abbreviations are used to describe peak patterns as appropriate: s = singlet, d = doublet, t = triplet, q = quartet, m = multiplet. Coupling constants J were reported in hertz. High-resolution mass spectra (HRMS) data were obtained with matrix-assisted laser desorption ionization time-of-flight (MLADI-TOF) mass spectrometer. Flash column chromatography was performed employing 300-400 mesh silica gel. Thin layer chromatography (TLC) was performed on silica gel HSGF254. The absorption spectra were measured using UV-vis spectrometer. FL spectra were recorded on a fluorospectro photometer with a xenon lamp excitation source. PL decay dynamics were measured on a time correlated single-photon counting (TCSPC) spectrofluorometer at room temperature, and the compounds were excited at its corresponding maximum absorption wavelength by picosecond laser diode. Cyclic voltammetry measurements were performed on an electrochemical analyzer in dichloromethane at room temperature. Thermogravimetric Analysis (TGA) was obtained with a thermal analyzer at heating and cooling rates of 10 K/min under an N_2 atmosphere. CLSM images were recorded with a Leica TCS SP5 confocal scanning system.

Device Fabrication: before device fabrication, the ITO glass substrates were pre-cleaned carefully. Then hole transporting material PEDOT: PSS was made with 4000 r/s for 45 s and annealing 25 min at 150°C. Emission Layer was prepared in chlorobenzene. Then it was made with 3000 r/s for 50 s and annealing 20 min at 100°C. After the organic film deposition, 40 nm of TPBi, 1.5 nm of LiF and 50 nm of aluminium were thermally evaporated onto the organic surface.

General procedure for synthesis of organic molecular

1. Synthesis of quinazolines **A** and **B**

A and **B** were synthesized according to reported literature[1].

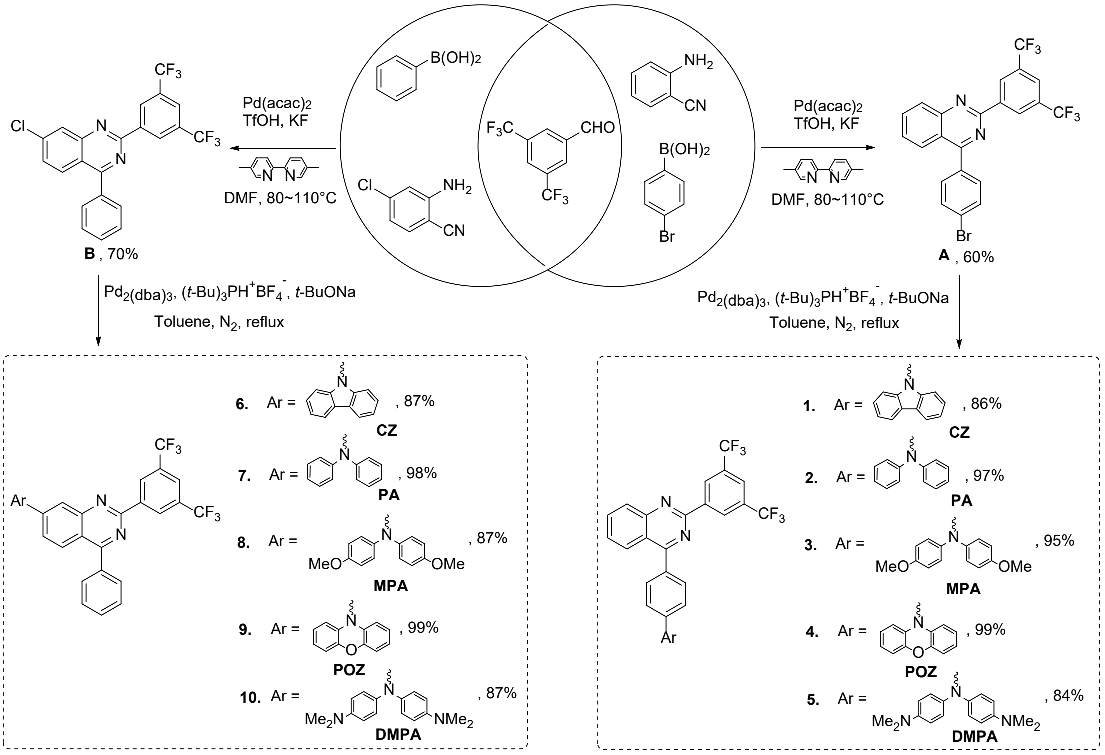

Figure S1 Synthetic route to target molecules **1-10**

2-Aminobenzonitriles (177mg, 1.5mmol), arylboronic acids (750mg, 3.75mmol), aldehydes (726mg, 3mmol), $Pd(acac)_2$ (5mol%), Ligand (10mol%) and KF (2equiv) were added into the schrenk tube (100mL). DMF (15mL) was added to dissolve the reactants. Stirring until the reactants completely dissolved, TfOH (4 equiv) was added. Keep the temperature at 80-110 °C for 48 h under air. After cooling to room temperature, the reaction mixture was poured into DCM, which was washed with saturated $NaHCO_3$ (2 × 50 mL) and then brine (1 ×50 mL). The organic layers were separated, dried over anhydrous Na_2SO_4 and evaporated under a vacuum. The residue was purified by flash column chromatography with petroleum ether: DCM = 4:1 to afford the desired product as a white solid, yield 60% for **A** and 70% for **B**, respectively.

Quinazoline **A**: ^{1}H NMR (400 MHz, Chloroform-*d*) δ 9.17 (s, 2H), 8.22 (d, *J* = 8.5 Hz, 1H), 8.12 (d, *J* = 8.4 Hz, 1H), 8.04 – 7.93 (m, 2H), 7.78 (s, 4H), 7.65 (t, *J* = 7.6 Hz, 1H). ^{13}C NMR (101 MHz, $CDCl_3$) δ 167.8, 157.2, 151.8, 140.1, 135.9, 134.3, 131.9 (q, *J* = 34.4 Hz), 132.0, 131.8, 129.5, 128.6, 128.6, 128.3, 126.8, 125.1, 123.9, 123.5 (q, *J* = 272.9 Hz), 121.9. ^{19}F NMR (376 MHz, $CDCl_3$) δ -62.67.

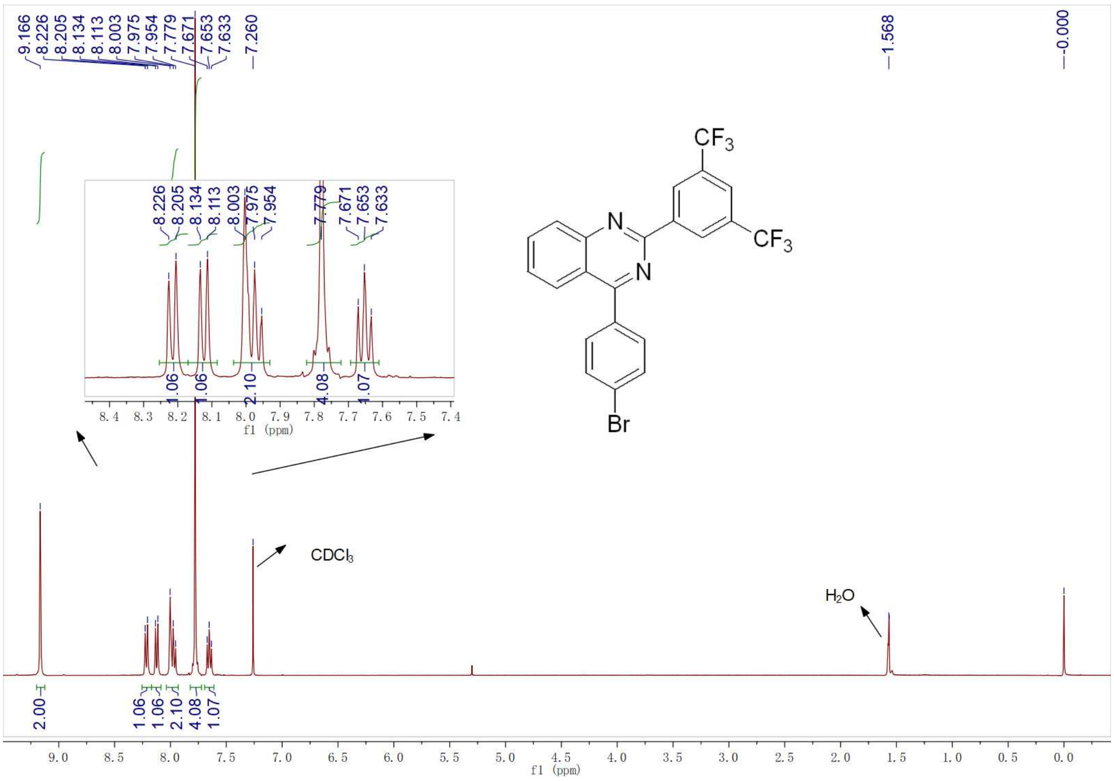

Figure S2 ^{1}H NMR of **A** in $CDCl_3$

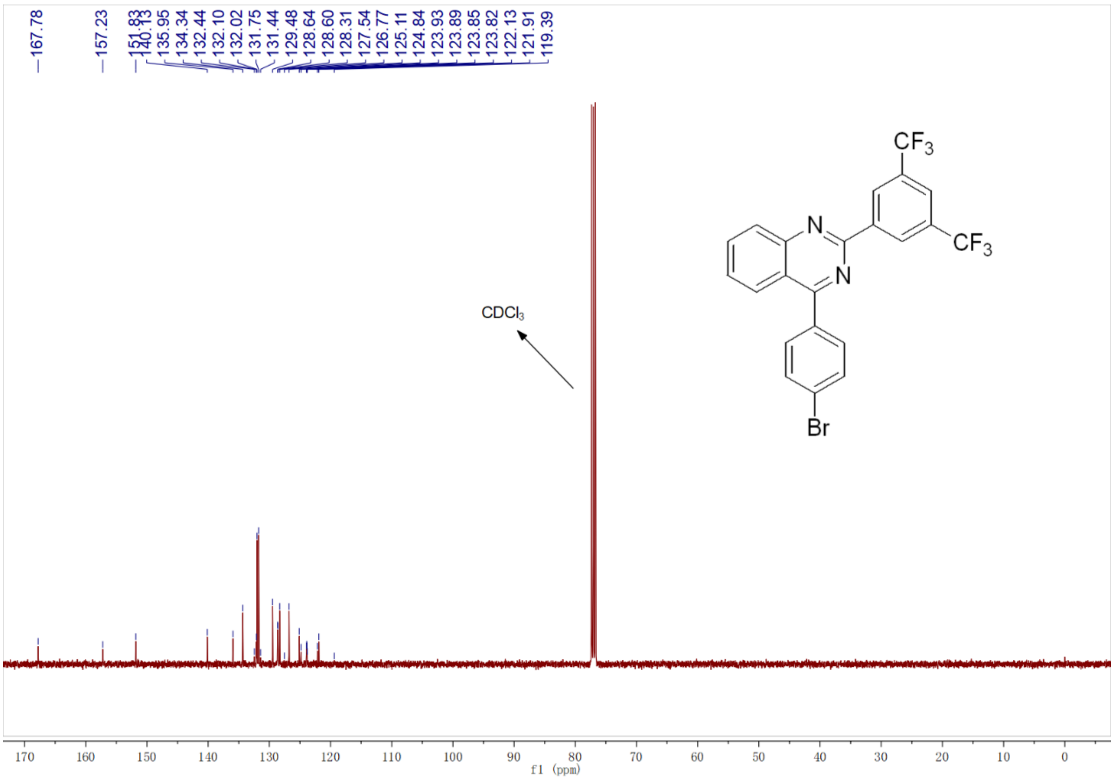

Figure S3 ^{13}C NMR of **A** in $CDCl_3$

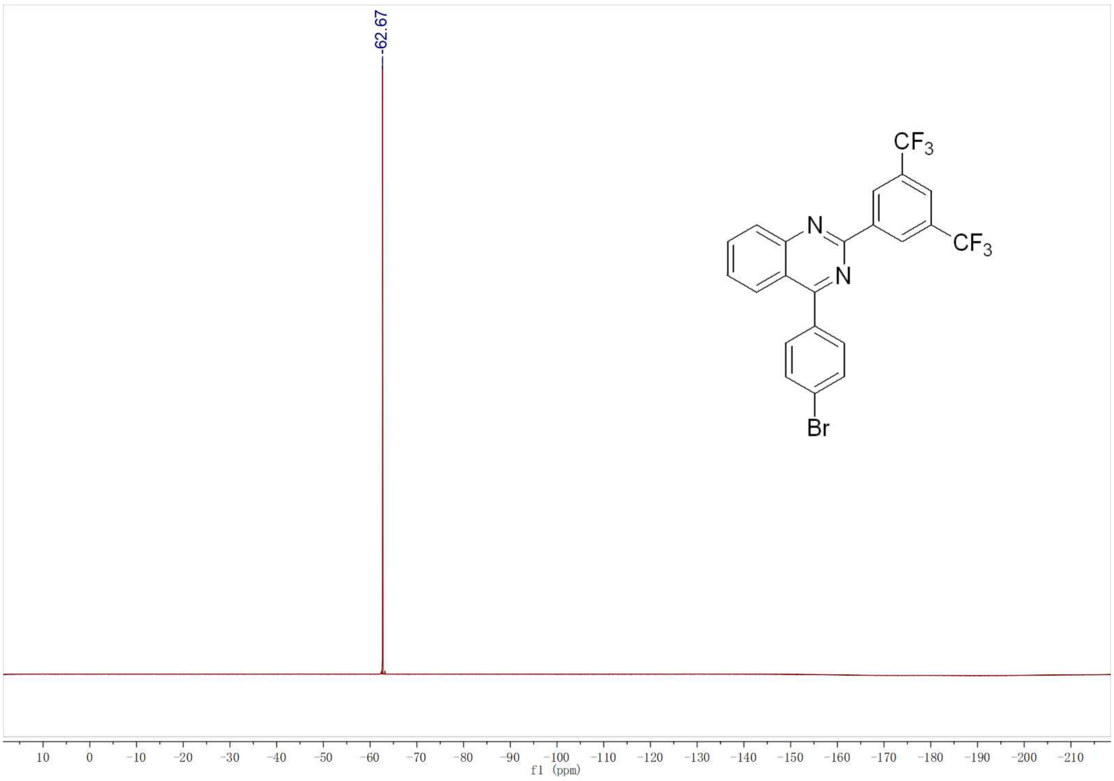

Figure S4 ^{19}F NMR of **A** in $CDCl_3$

Quinazoline **B**: ^{1}H NMR (400 MHz, Chloroform-*d*) δ 9.16 (s, 2H), 8.21 (s, 1H), 8.11 (d, *J* = 8.9 Hz, 1H), 8.01 (s, 1H), 7.91 – 7.82 (m, 2H), 7.71 – 7.60 (m, 3H), 7.57 (d, *J* = 11.0 Hz, 1H). ^{13}C NMR (101 MHz, $CDCl_3$) δ 168.9, 158.1, 152.4, 140.5, 139.9, 136.7, 131.9 (q, *J* = 33.4 Hz), 130.6, 130.1, 129.1, 128.8, 128.7, 128.7, 128.3, 124.0, 123.4 (q, *J* = 272.9 Hz), 120.5. ^{19}F NMR (376 MHz, $CDCl_3$) δ -62.72.

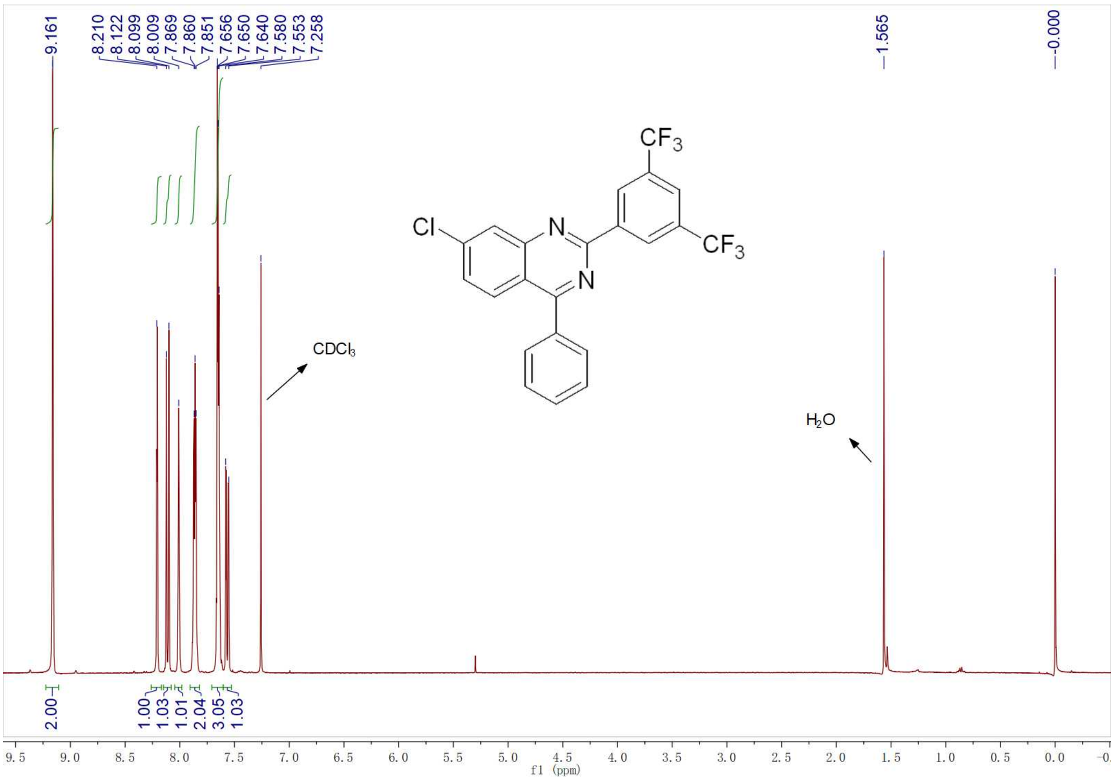

Figure S5 ^{1}H NMR of **B** in $CDCl_3$

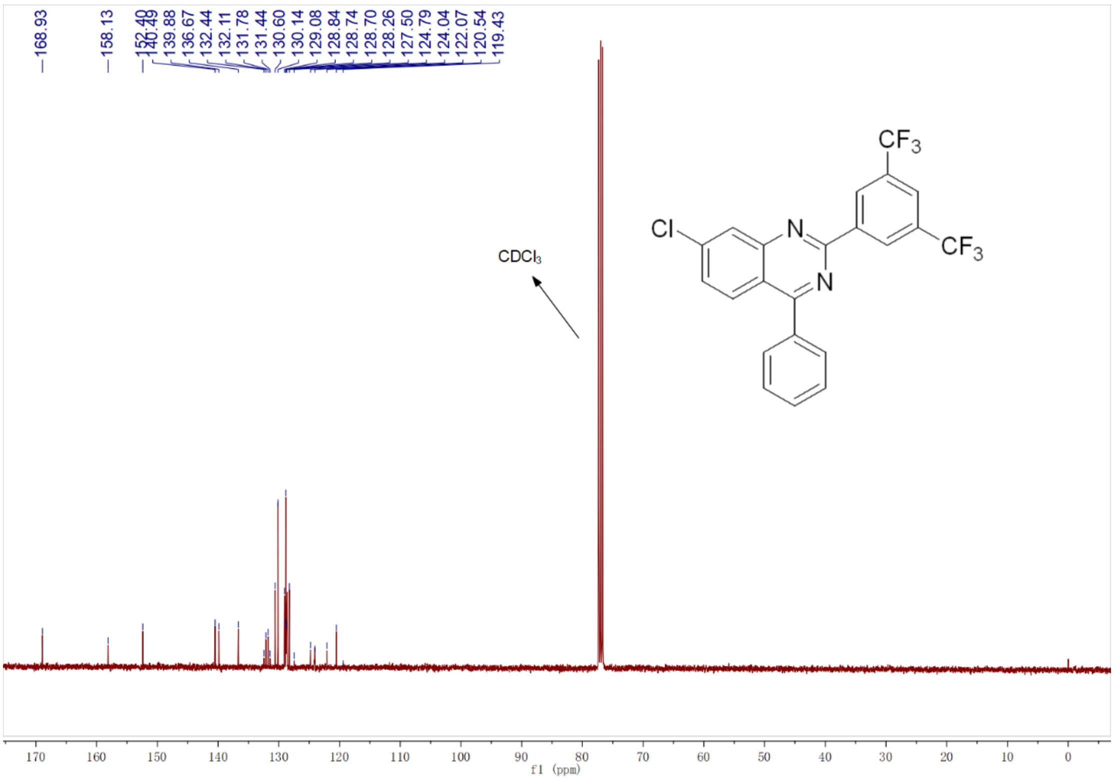

Figure S6 ^{13}C NMR of **B** in $CDCl_3$

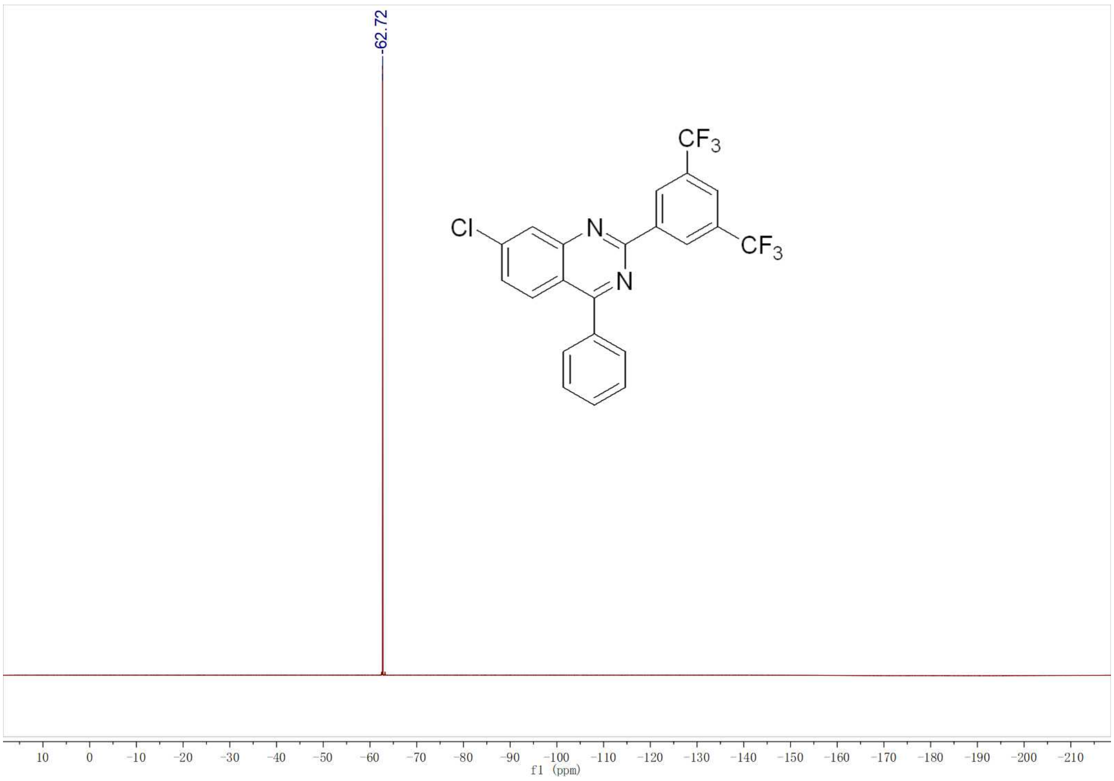

Figure S7 ^{19}F NMR of **B** in $CDCl_3$

2. Synthesis of the target molecules (**1**-**10**)

The target molecules (**1**-**10**) were synthesized according to Buchwald-Hartwig coupling.

The quinazoline A or B (100 mg, 1equiv) was added into the schrenk tube (25mL). Then the aromatic secondary amine (1.2equiv), $Pd_2(dba)_3$ (0.1equiv), $(t\text{-Bu})_3PH^+BF_4^-$ (0.3equiv) and *t*-BuONa (2equiv) were added. After replacing the air with N_2, toluene was added to dissolve the reactants. The mixture was stirred and refluxed for 24h under N_2. After cooling to room temperature, the solvent was removed via vacuum. The residue was purified by flash column chromatography with petroleum ether: DCM = 4:1 to 1:2, which depends on different kinds of amine, to afford the desired products 1-10.

Compound 1: DCM: PE = 1:4 as eluent, light cyan solid, yield 86.2%, mp 267-268 ^{o}C. ^{1}H NMR (400 MHz, $CDCl_3$) δ 9.25 (s, 2H), 8.34 (d, *J* = 8.3 Hz, 1H), 8.27 (d, *J* = 8.4 Hz, 1H), 8.18 (m, 4H), 8.02 (d, *J* = 10.6 Hz, 2H), 7.89 (d, *J* = 8.3 Hz, 2H), 7.73 (m, 1H), 7.63 (d, *J* = 8.2 Hz, 2H), 7.49 (m, 2H), 7.35 (m, 2H).^{13}C NMR (101 MHz, $CDCl_3$) δ 167.9, 157.4, 152.0, 140.6, 140.3, 139.9, 135.8, 134.4, 132.0 (q, *J* = 33.2 Hz), 131.9, 129.6, 128.7, 128.4, 127.0, 126.2, 124.0, 123.8, 123.6 (q, *J* = 273.1 Hz), 122.1, 120.5, 109.9. ^{19}F NMR (376 MHz, $CDCl_3$) δ -62.60. HRMS (Maldi-TOF): cacld. for $C_{34}H_{19}F_6N_3$ $[M+H]^+$, 583.1483; found: 583.1478. IR: 3084, 3062, 1607, 1451, 1311, 1277, 1174, 1126, 842, 774, 682.

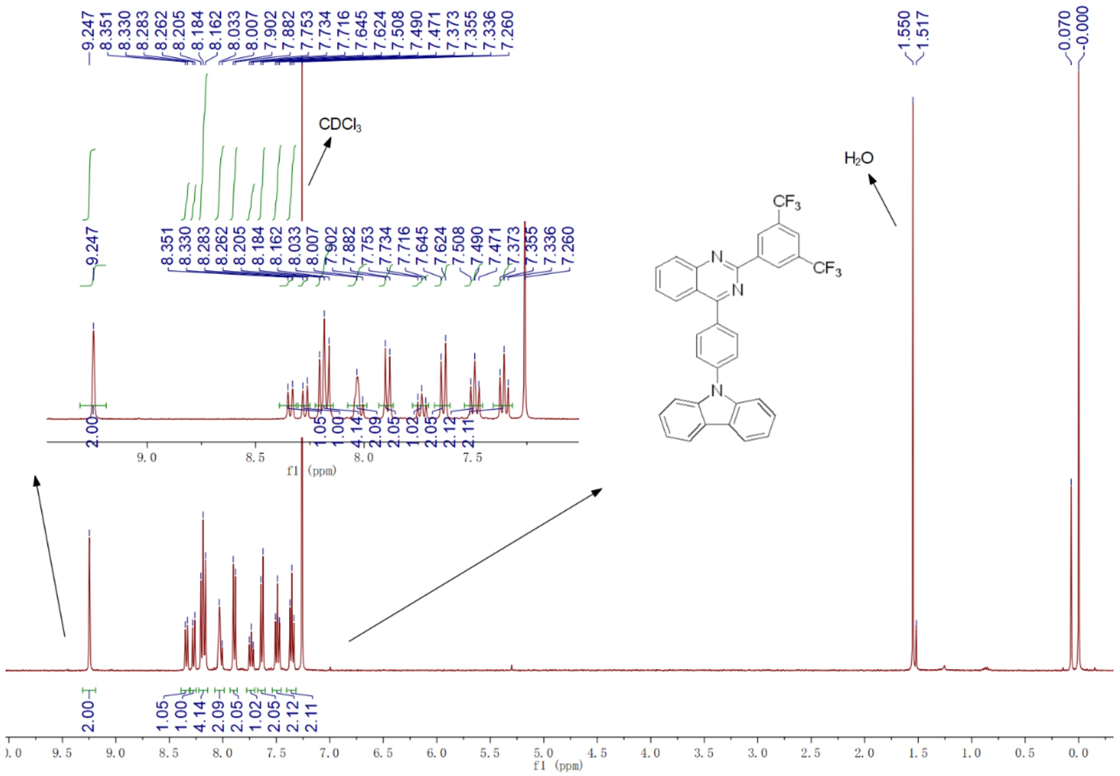

Figure 8 ^{1}H NMR of **1** in $CDCl_3$

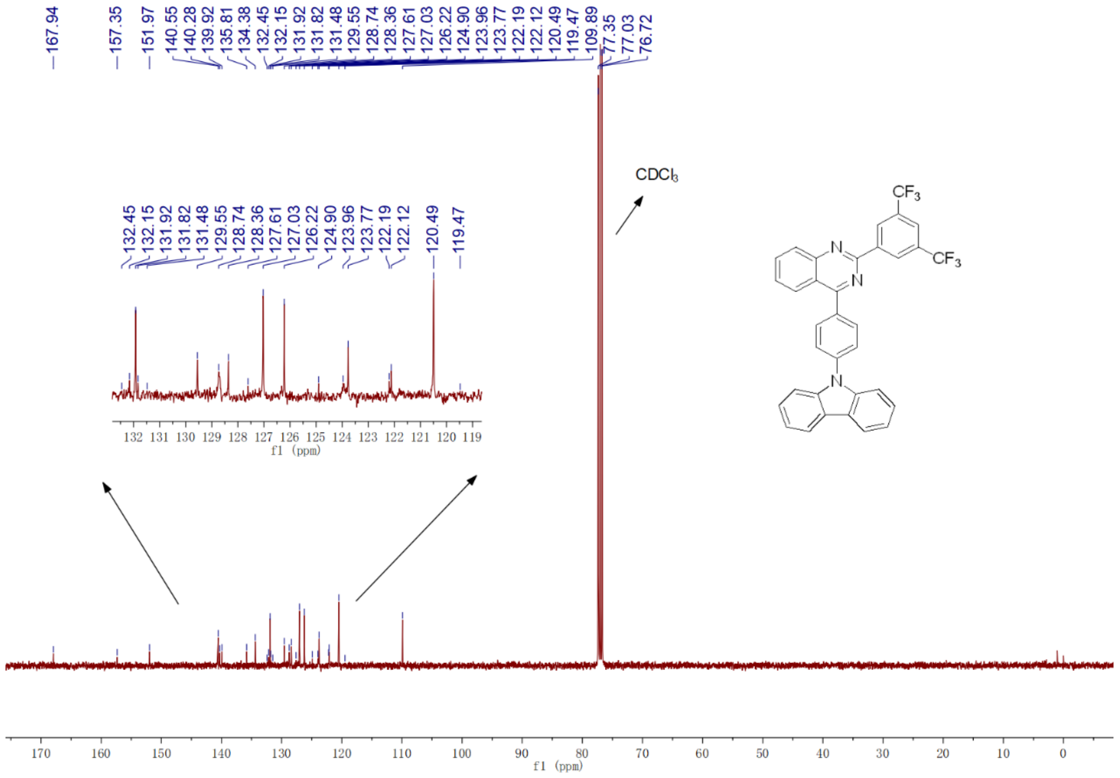

Figure S9 ^{13}C NMR of **1** in $CDCl_3$

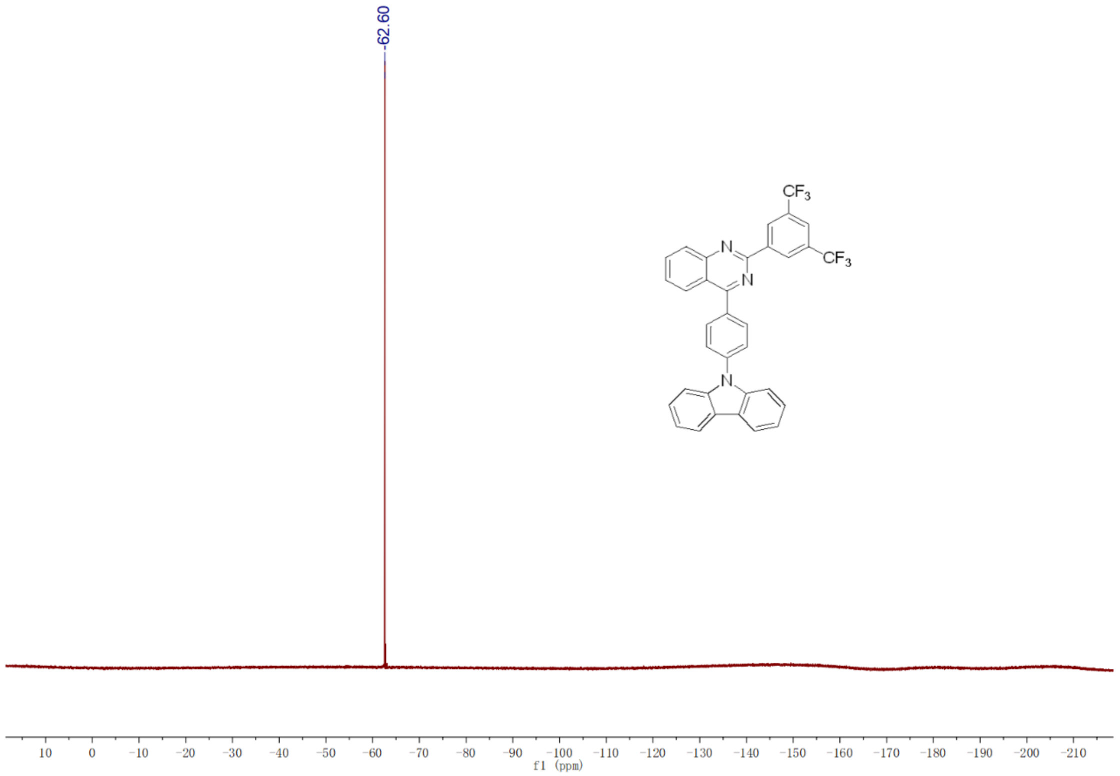

Figure 10 ^{19}F NMR of **1** in $CDCl_3$

Compound 2: DCM:PE=1:4 as eluent, green yellow solid, yield 97.2%, mp 197-198 ^{o}C.^{1}H NMR (400 MHz, $CDCl_3$) δ 9.19 (s, 2H), 8.30 (d, *J* = 8.3 Hz, 1H), 8.18 (d, *J* = 8.4 Hz, 1H), 7.99 (s, 1H), 7.94 (m, 1H), 7.82 (d, *J* = 8.6 Hz, 2H), 7.64 (m, 1H), 7.36 (m, 4H), 7.24 (d, *J* = 9.4 Hz, 6H), 7.14 (m, 2H).^{13}C NMR (101 MHz, $CDCl_3$) δ 168.1, 157.2, 151.9, 150.3, 147.0, 140.5, 133.9, 131.8 (q, *J* = 33.2 Hz), 131.6, 129.6, 129.3, 128.7, 127.8, 127.4, 125.6, 123.7, 123.6 (q, *J* = 272.6 Hz), 122.0, 121.4.^{19}F NMR (376 MHz, $CDCl_3$) δ -62.62.HRMS (Maldi-TOF): cacld. for $C_{34}H_{21}F_6N_3[M+H]^+$,

585.1640; found: 585.1635. IR:3058, 3037, 1591, 1491, 1313, 1286, 1172, 1132, 700, 682.

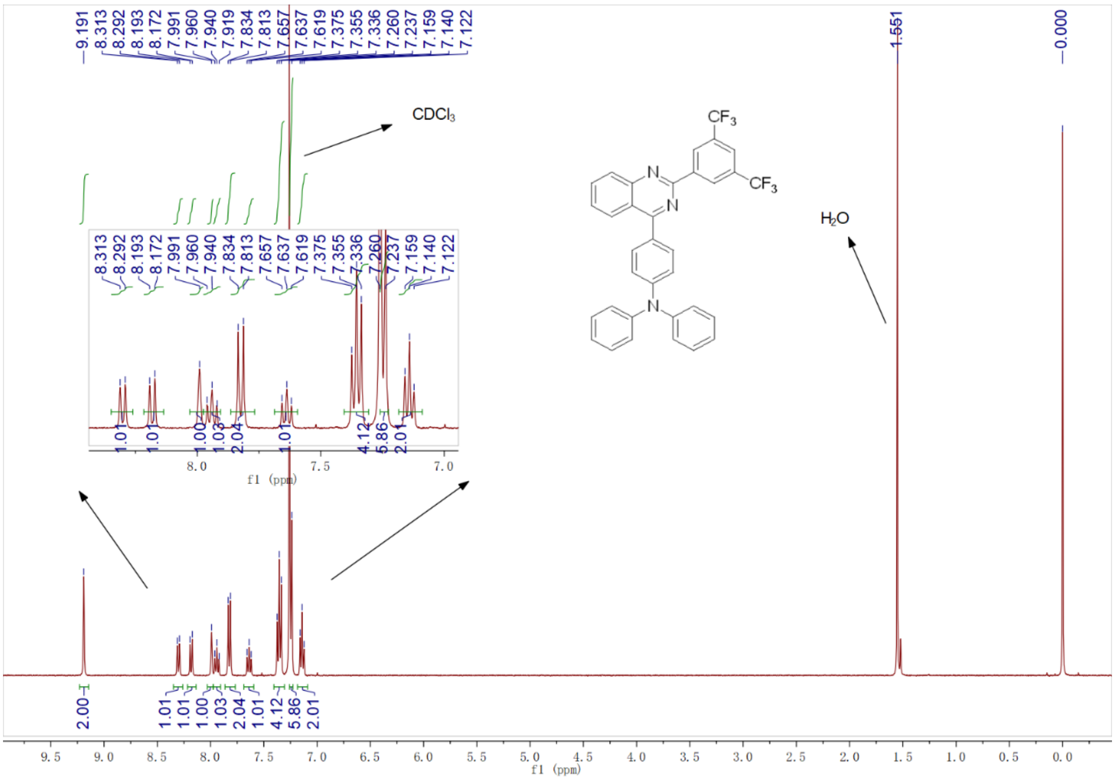

Figure 11 ^{1}H NMR of **2** in $CDCl_3$

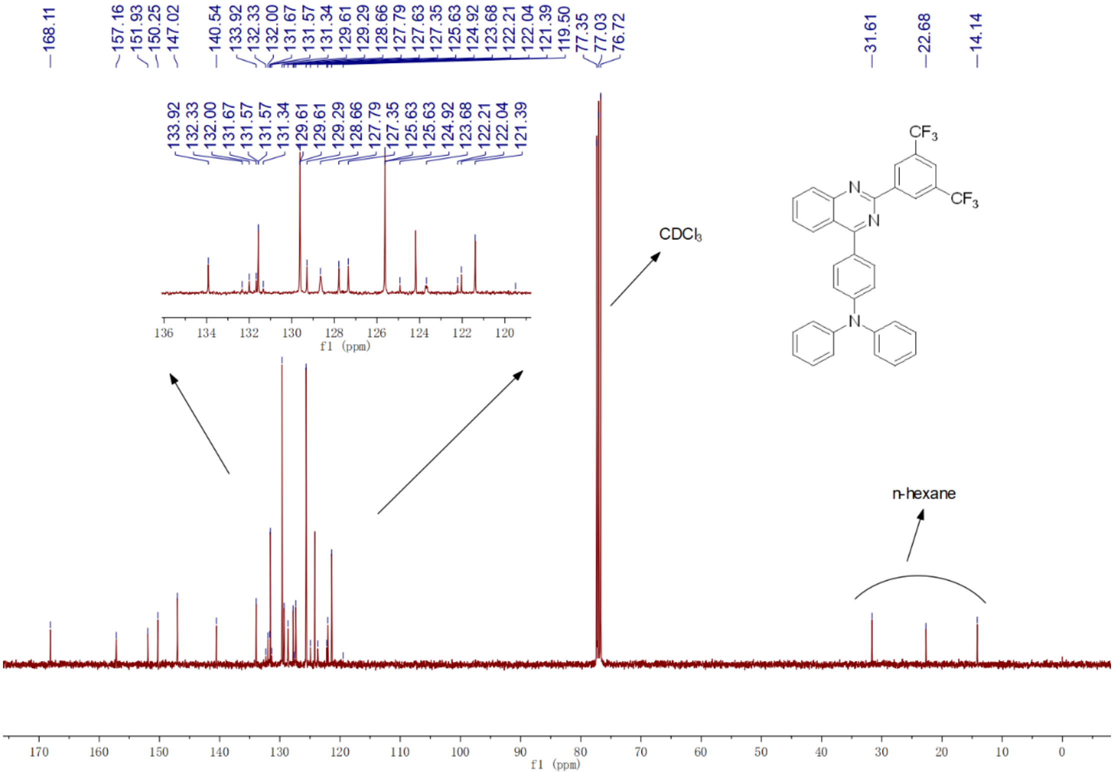

Figure 12 ^{13}C NMR of **2** in $CDCl_3$

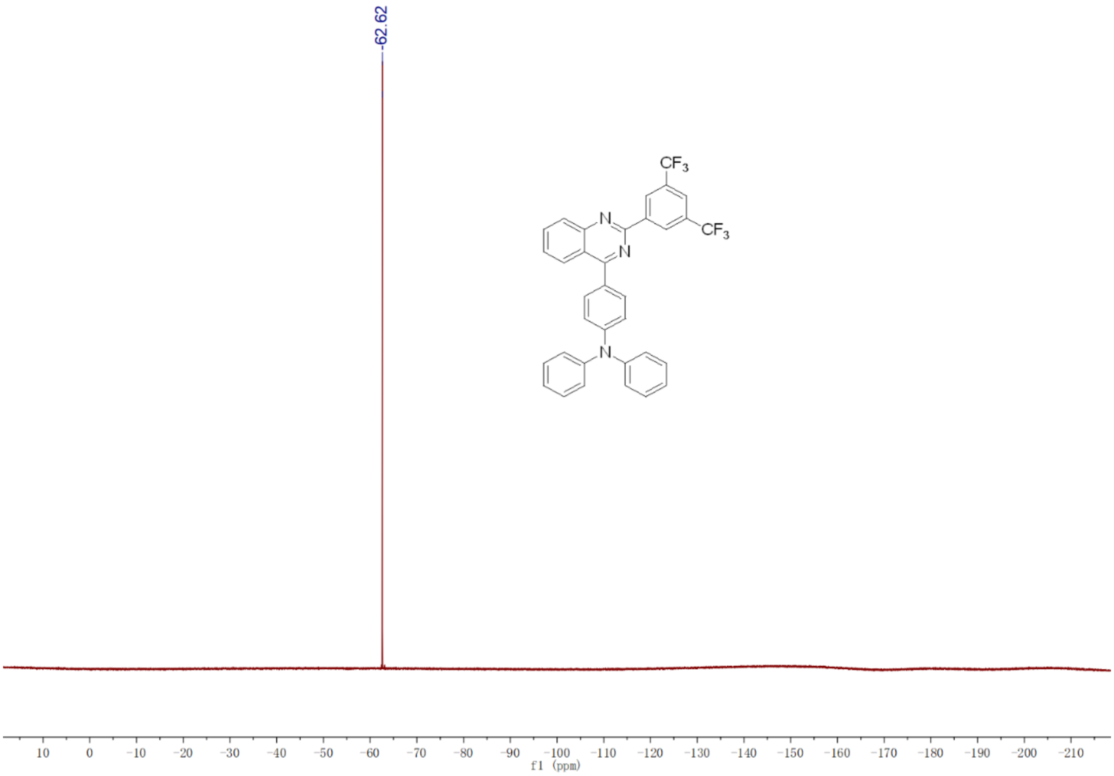

Figure S13 ^{19}F NMR of **2** in $CDCl_3$

Compound 3: DCM:PE=1:4 as eluent, orange solid, yield 94.7%, mp 190-191°C.^{1}H NMR (400 MHz, $CDCl_3$) δ 9.19 (s, 2H), 8.33 – 8.26 (m, 1H), 8.16 (d, *J* = 8.3 Hz, 1H), 7.98 (s, 1H), 7.92 (m, 1H), 7.79 (d, *J* = 8.7 Hz, 2H), 7.61 (m, 1H), 7.20 (d, *J* = 8.9 Hz, 4H), 7.08 (d, *J* = 8.7 Hz, 2H), 6.92 (d, *J* = 8.9 Hz, 4H), 3.83 (s, 6H).^{13}C NMR (101 MHz, $CDCl_3$) δ 168.2, 157.1, 156.8, 151.9, 151.1, 140.6, 139.8, 133.8, 131.8 (q, *J* = 33.5 Hz), 131.6, 129.2, 128.6, 127.7, 127.4, 123.6 (q, *J* = 273.5 Hz), 123.6, 122.0, 118.4, 115.0, 55.5.^{19}F NMR (376 MHz, $CDCl_3$) δ -62.62.HRMS (Maldi-TOF): cacld. for $C_{36}H_{25}F_6N_3O_2$[M+H]$^+$, 645.1851; found: 645.1848. IR:2942, 2830, 1601, 1505, 1279, 1244. 1173, 1133, 829, 700, 682.

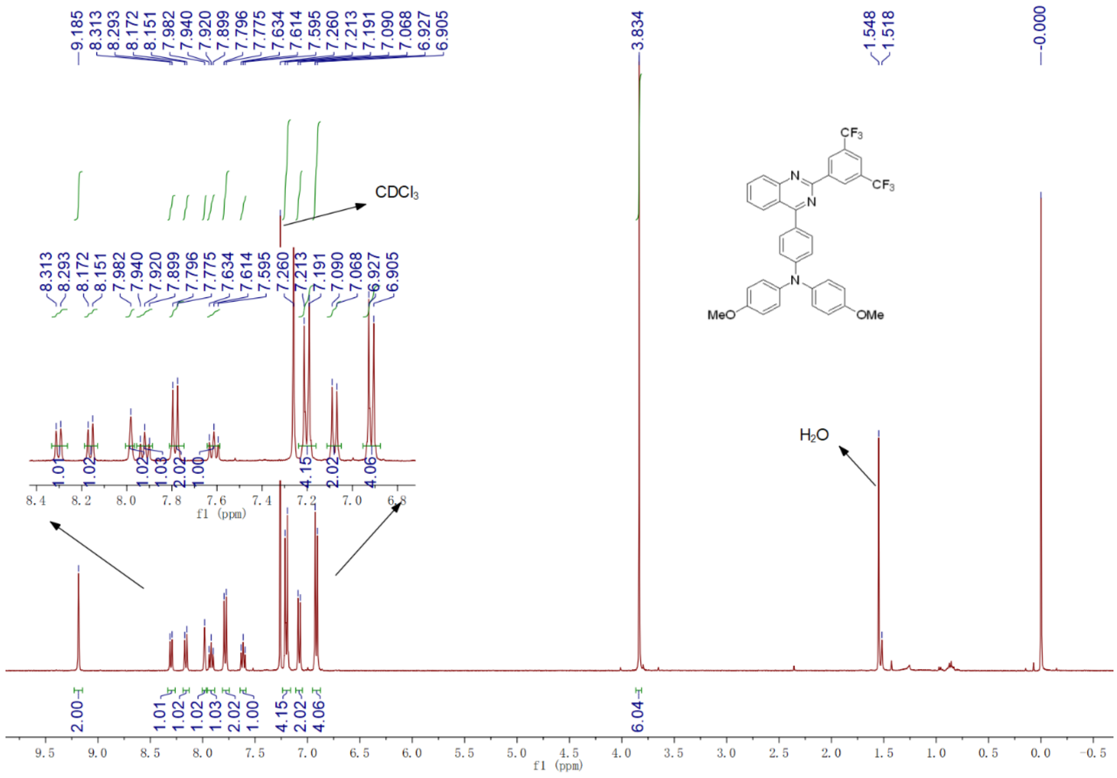

Figure 14 ^{1}H NMR of **3** in $CDCl_3$

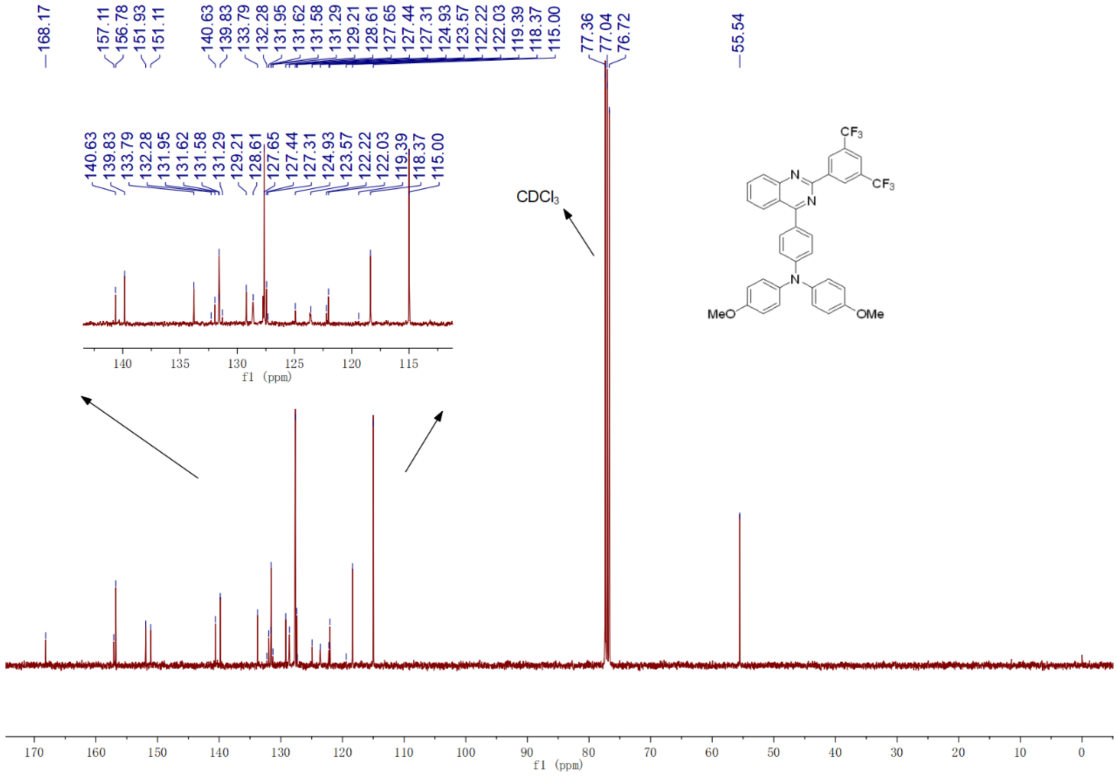

Figure S15 ^{13}C NMR of **3** in $CDCl_3$

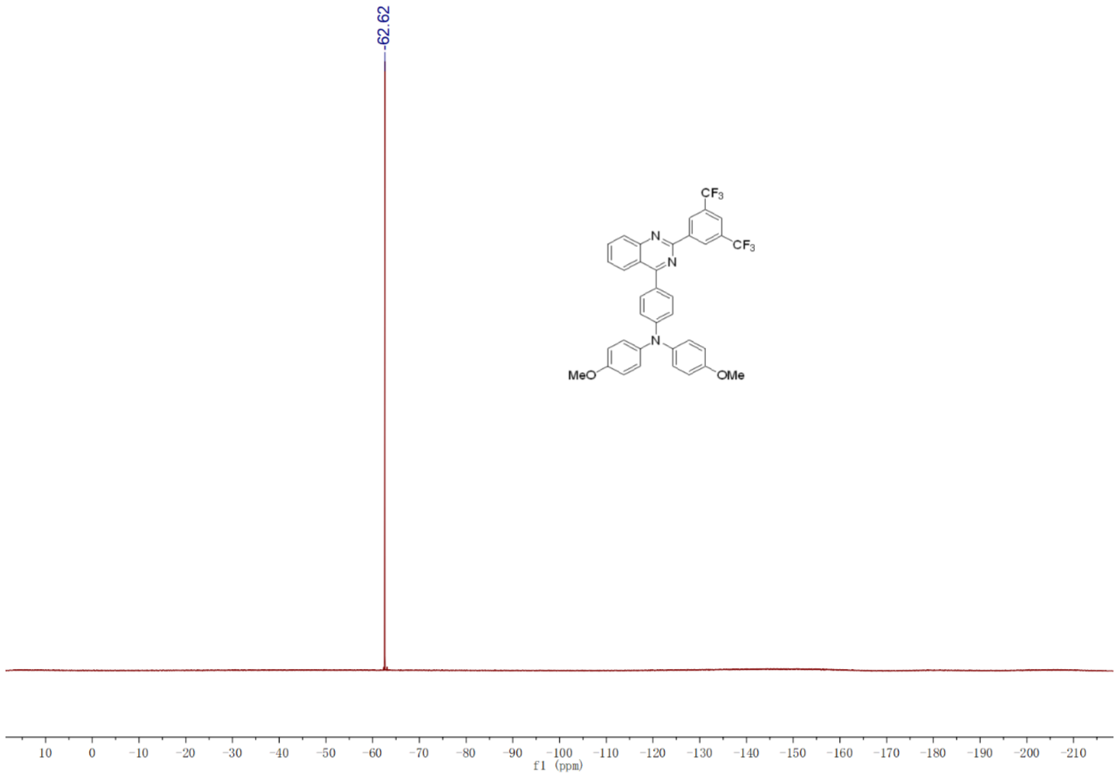

Figure S16 ^{19}F NMR of **3** in $CDCl_3$

Compound 4: DCM:PE=1:4 as eluent, yellow solid, yield >99%, mp 245-246 °C.^{1}H NMR (400 MHz, $CDCl_3$) δ 9.23 (s, 2H), 8.26 (d, *J* = 8.6 Hz, 2H), 8.14 (d, *J* = 8.3 Hz, 2H), 8.01 (d, *J* = 12.6 Hz, 2H), 7.72 (m, 1H), 7.64 (d, *J* = 8.3 Hz, 2H), 6.78 – 6.65 (m, 6H), 6.13 (dd, *J* = 7.4, 1.9 Hz, 2H).^{13}C NMR (101 MHz, $CDCl_3$) δ 167.9, 157.3, 151.9, 144.1, 141.2, 140.2, 137.1, 134.4, 134.1, 133.0, 132.0 (q, *J* = 33.3 Hz), 131.3, 129.6, 129.1, 128.7, 128.4, 126.9, 123.9, 123.6 (q, *J* = 272.6 Hz), 123.4, 122.0, 121.8, 115.7, 113.5.^{19}F NMR (376 MHz, $CDCl_3$) δ -62.62.HRMS (Maldi-TOF): cacld. for $C_{34}H_{19}F_6N_3O$ $[M+H]^+$, 599.1432; found: 599.1430. IR:3067, 2920, 1603, 1487, 1315, 1278, 1170, 1134, 744, 701, 682.

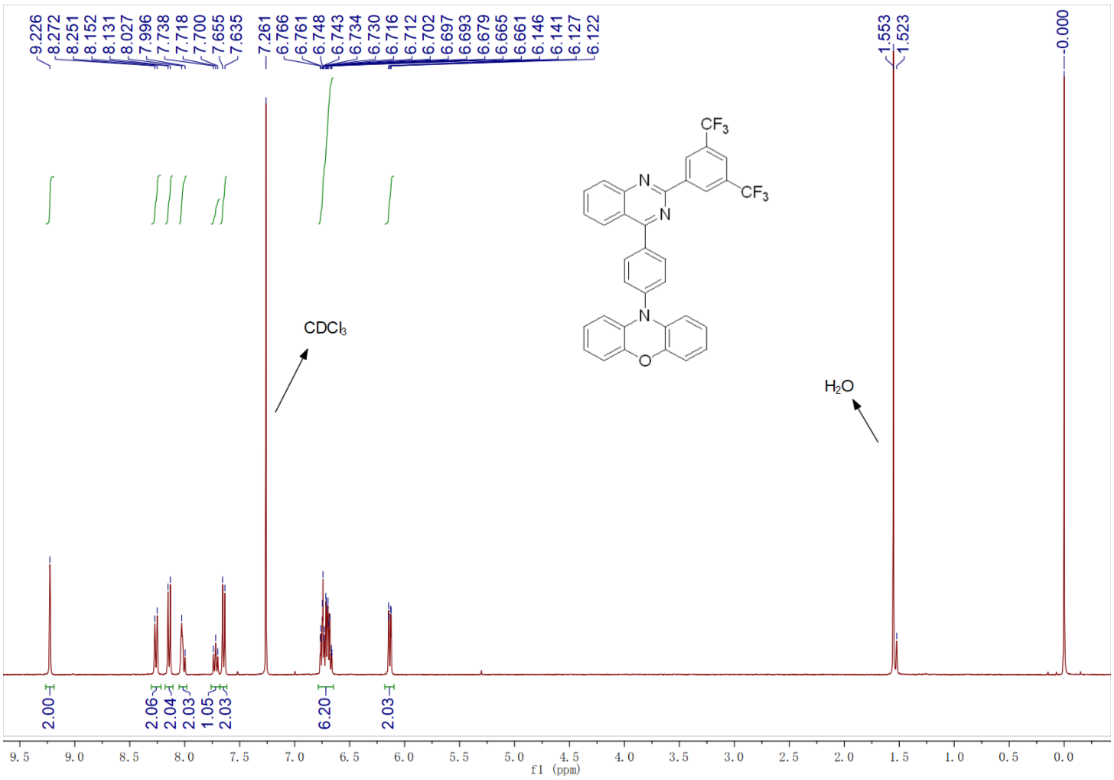

Figure S17 ^{1}H NMR of **4** in $CDCl_3$

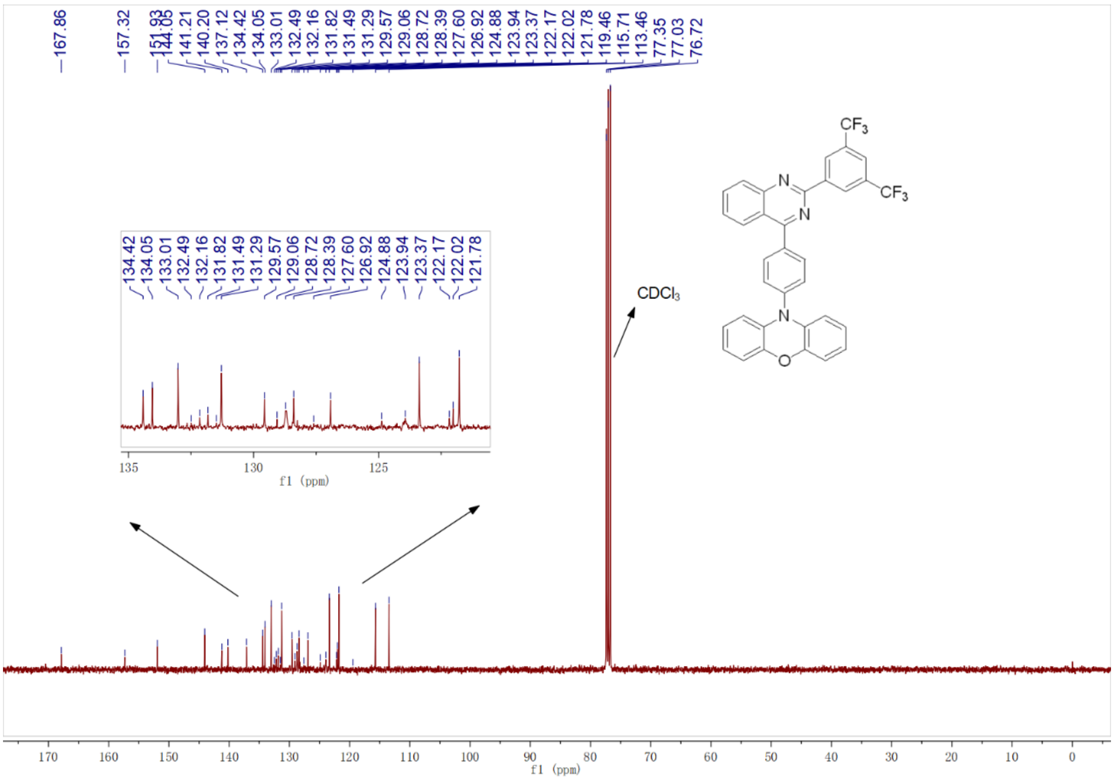

Figure S18 ^{13}C NMR of **4** in $CDCl_3$

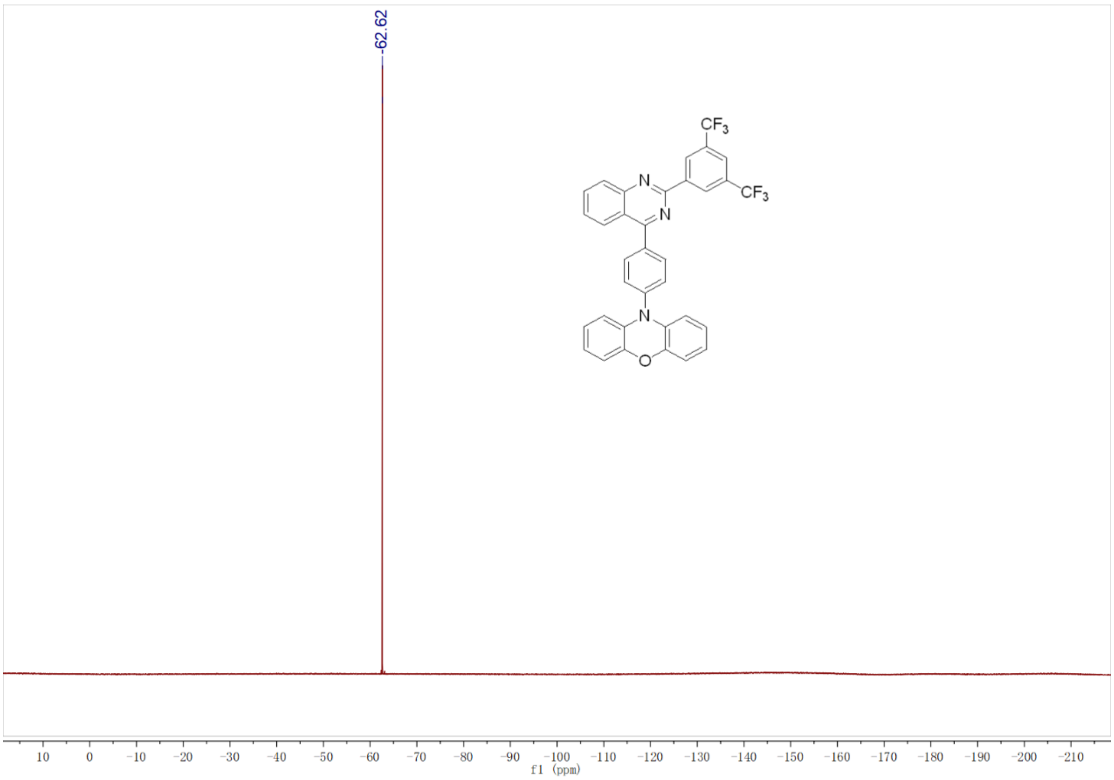

Figure S19 ^{19}F NMR of **4** in $CDCl_3$

Compound 5: DCM:PE=2:1 as eluent, red solid, yield 83.7%, mp 212-213 °C.^{1}H NMR (400 MHz, $CDCl_3$) δ 9.19 (s, 2H), 8.32 (d, *J* = 8.0 Hz, 1H), 8.14 (d, *J* = 8.3 Hz, 1H), 7.97 (s, 1H), 7.90 (m, 1H), 7.77 (s, 2H), 7.59 (m, 1H), 7.17 (s, 6H), 6.75 (s, 4H), 2.97 (s, 12H).^{13}C NMR (101 MHz, $CDCl_3$) δ 157.1, 151.9, 140.7, 133.7, 131.7 (q, *J* = 33.6 Hz), 129.1, 128.6, 127.6, 127.5, 123.6 (q, *J* = 272.9 Hz), 123.5, 122.0, 113.7.^{19}F NMR (376 MHz, $CDCl_3$) δ -62.61.HRMS (Maldi-TOF): cacld. for $C_{38}H_{31}F_6N_5$ $[M+H]^+$, 671.2484; found: 671.2482. IR:3032, 2882, 1600, 1513, 1385, 1286, 1171, 1132, 812, 770, 683.

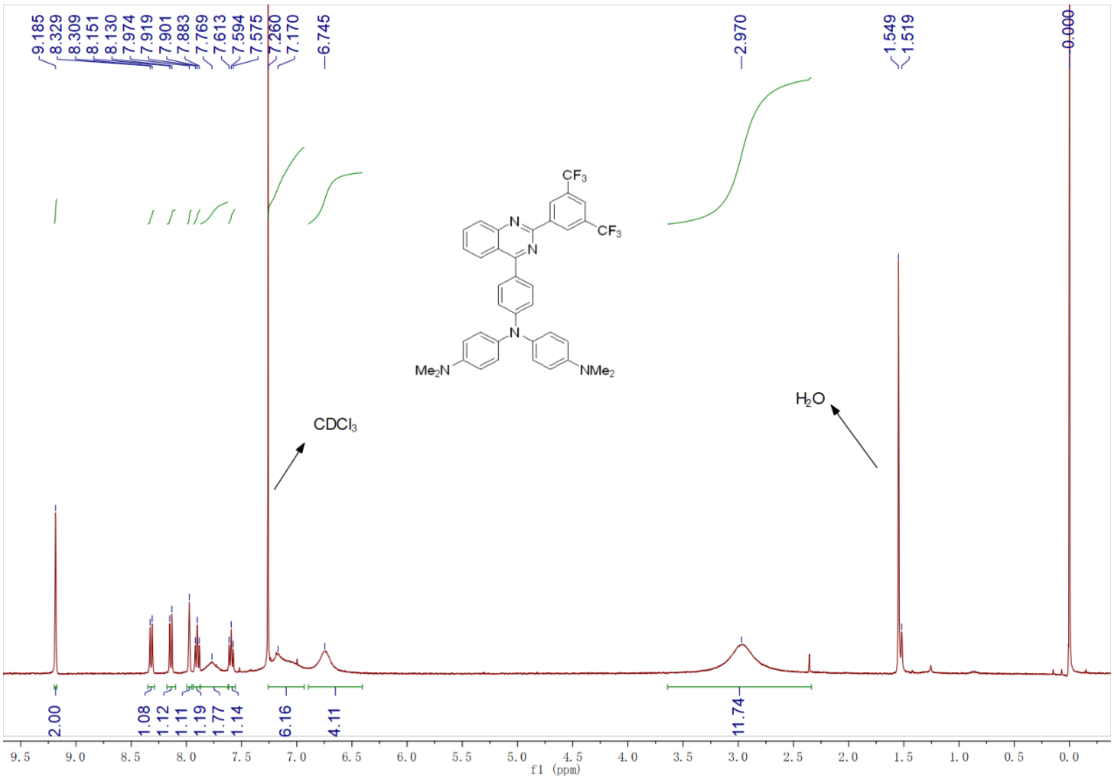

Figure S20 ^{1}H NMR of **5** in $CDCl_3$

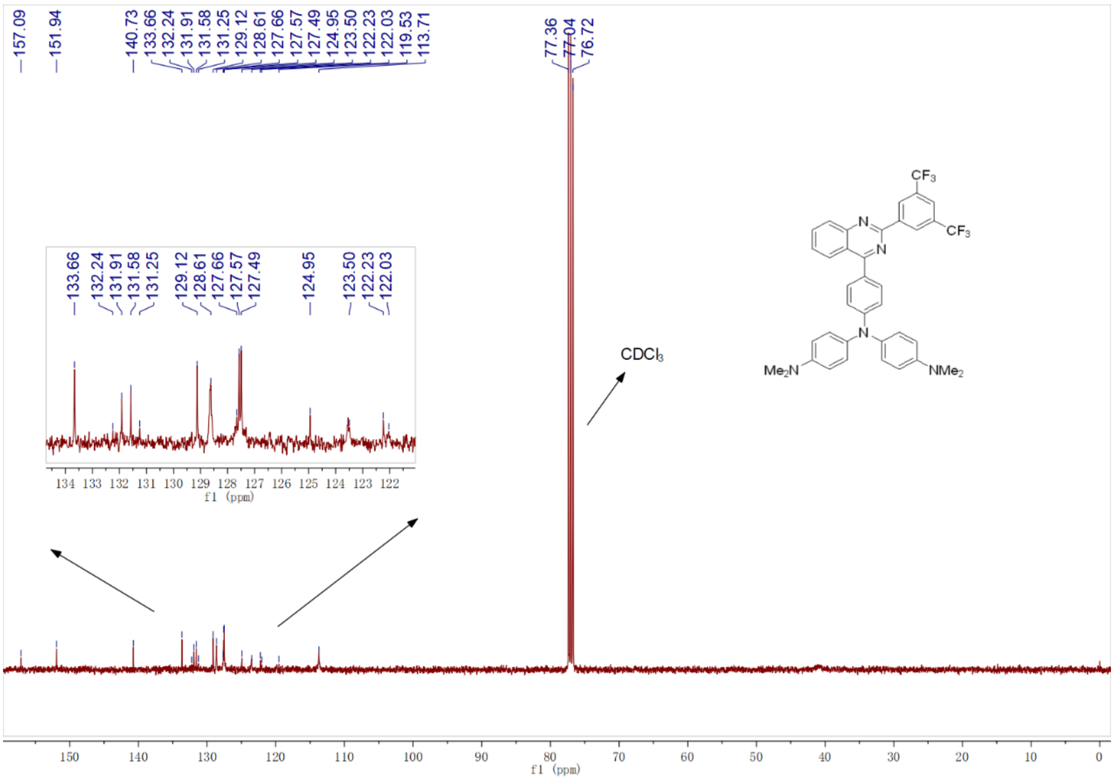

Figure S21 ^{13}C NMR of **5** in $CDCl_3$

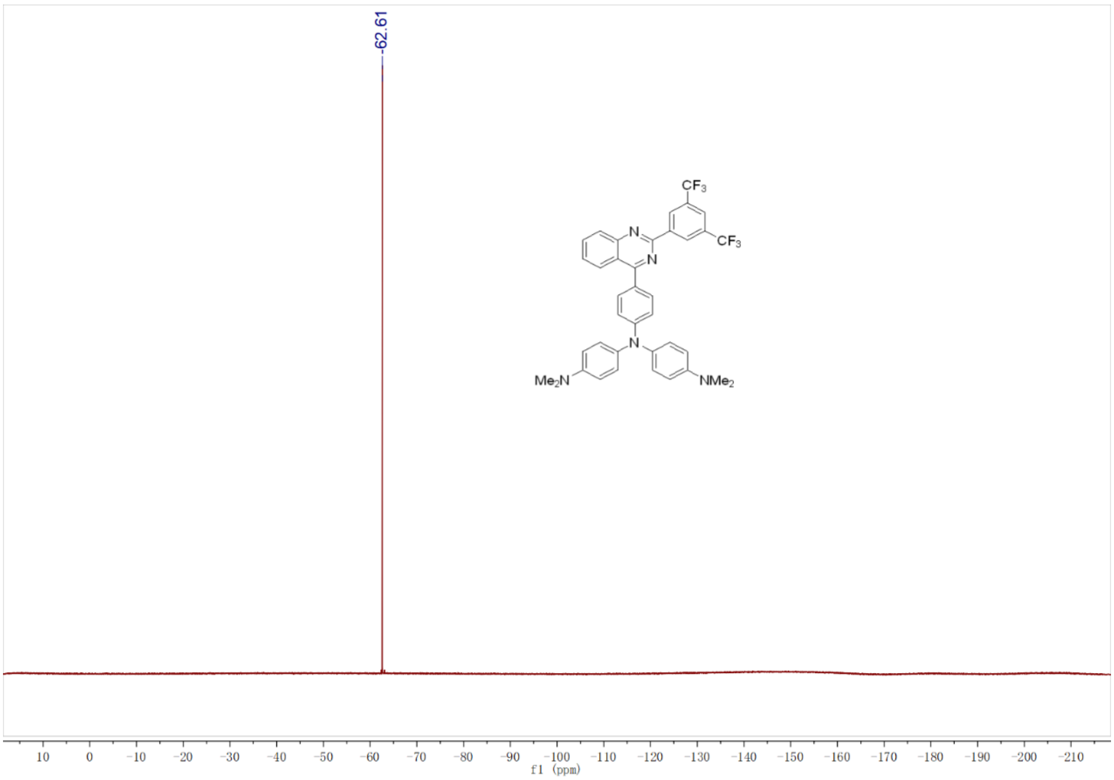

Figure S22 ^{19}F NMR of **5** in $CDCl_3$

Compound 6: DCM:PE=1:4 as eluent, light cyan solid, yield 86.6%, mp 304-305 °C. ^{1}H NMR (400 MHz, $CDCl_3$) δ 9.23 (s, 2H), 8.45 (d, *J* = 1.9 Hz, 1H), 8.41 (d, *J* = 8.9 Hz, 1H), 8.19 (d, *J* = 7.7 Hz, 2H), 8.03 (s, 1H), 7.98 (dd, *J* = 6.5, 3.1 Hz, 2H), 7.93 (dd, *J* = 8.9, 2.1 Hz, 1H), 7.68 (dd, *J* = 5.3, 3.1 Hz, 5H), 7.49 (m, 2H), 7.38 (m, 2H). ^{13}C NMR (101 MHz, $CDCl_3$) δ 168.8, 158.2, 153.1, 143.2, 140.1, 136.9, 132.0 (q, *J* = 33.5 Hz), 130.6, 130.2, 129.2, 128.9, 126.7, 126.5, 125.1, 123.5 (q, *J* = 273.2 Hz), 124.2, 124.1, 121.1, 120.7, 120.6, 116.9, 109.9.^{19}F NMR (376 MHz, $CDCl_3$) δ -62.64. HRMS (Maldi-TOF): cacld. for $C_{34}H_{19}F_6N_3$ $[M+H]^+$, 583.1483; found: 583.1479. IR:3075, 2925, 1615, 1493, 1448, 1327, 1281, 1130, 748, 723, 701, 682.

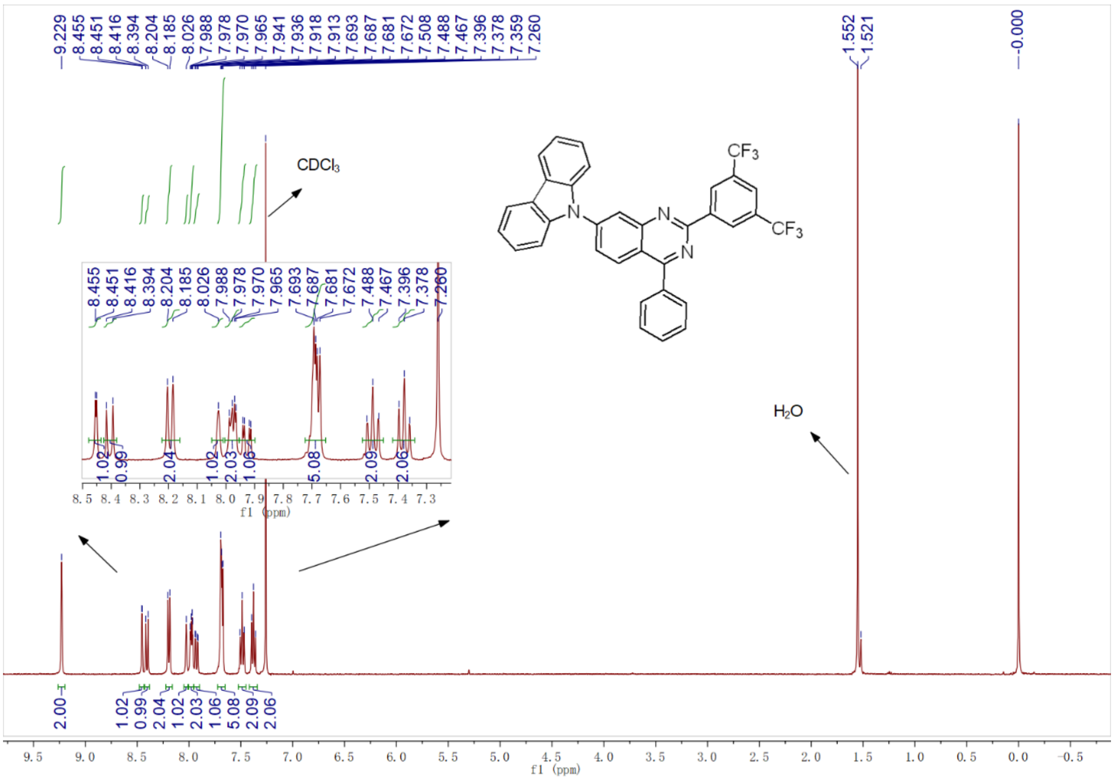

Figure S23 ^{1}H NMR of **6** in $CDCl_3$

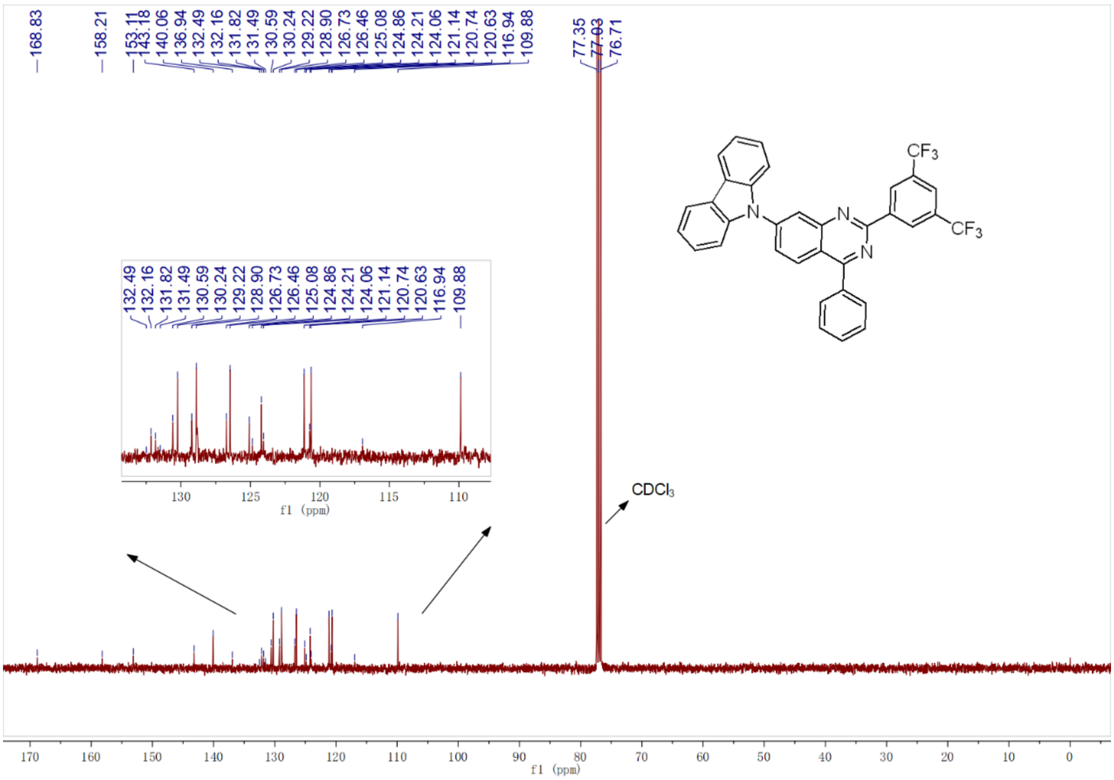

Figure S24 ^{13}C NMR of **6** in $CDCl_3$

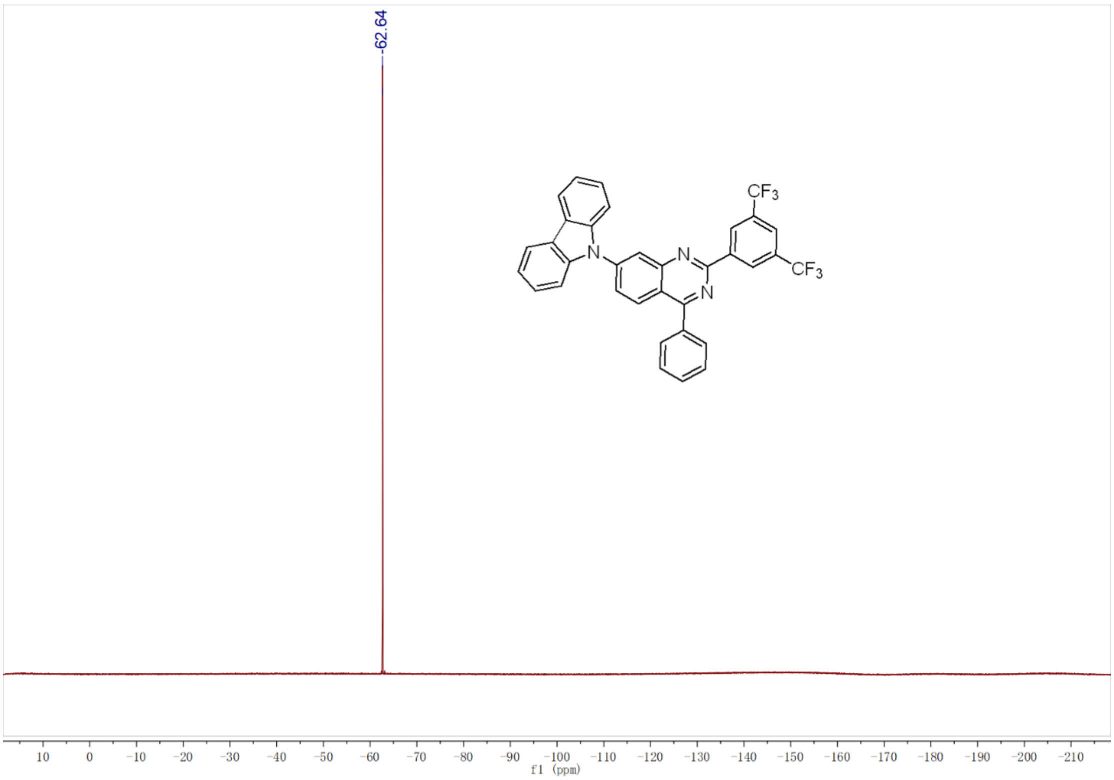

Figure S25 ^{19}F NMR of **6** in $CDCl_3$

Compound 7: DCM:PE=1:4 as eluent, yellow solid, yield 97.7%, mp 278-279 ^{o}C. ^{1}H NMR (400 MHz, $CDCl_3$) δ 9.09 (s, 2H), 7.95 (s, 1H), 7.92 (d, *J* = 9.3 Hz, 1H), 7.86 (dd, *J* = 6.6, 3.0 Hz, 2H), 7.63 – 7.55 (m, 3H), 7.46 (d, *J* = 2.3 Hz, 1H), 7.40 (m, 4H), 7.30 (d, *J* = 2.4 Hz, 1H), 7.27 (s, 3H), 7.22 (d, *J*= 7.3 Hz, 3H).^{13}C NMR (101 MHz, $CDCl_3$) δ 167.2, 157.9, 153.6, 152.8, 146.0, 140.7, 137.5, 131.7 (q, *J* = 33.5 Hz), 130.1, 129.9, 128.6, 127.9, 126.5, 125.5, 123.6 (q, *J* = 273.1 Hz), 123.5, 122.9, 117.2, 114.4.^{19}F NMR (376MHz, $CDCl_3$) δ -62.60.HRMS (Maldi-TOF): cacld. for $C_{34}H_{21}F_6N_3$ $[M+H]^+$, 585.1640; found: 585.1639. IR:3092, 3062, 1595, 1485, 1406, 1326, 1286, 1170, 1131, 753, 700, 683.

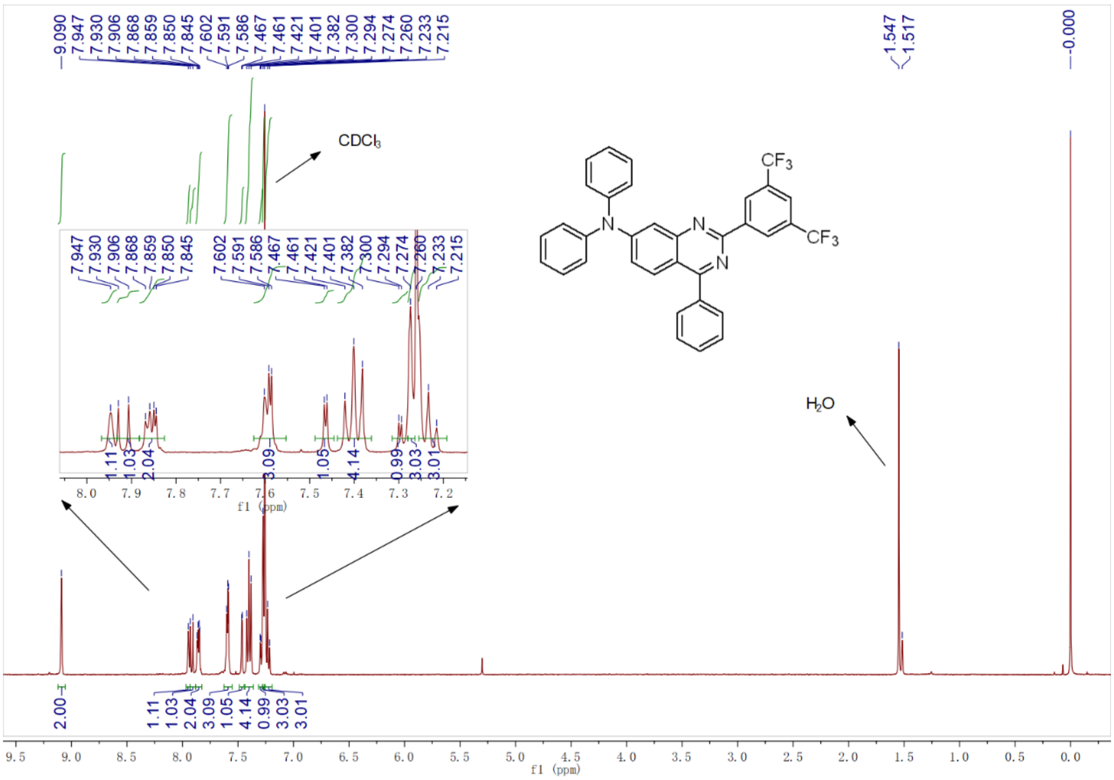

Figure S26 ^{1}H NMR of **7** in CDCl3

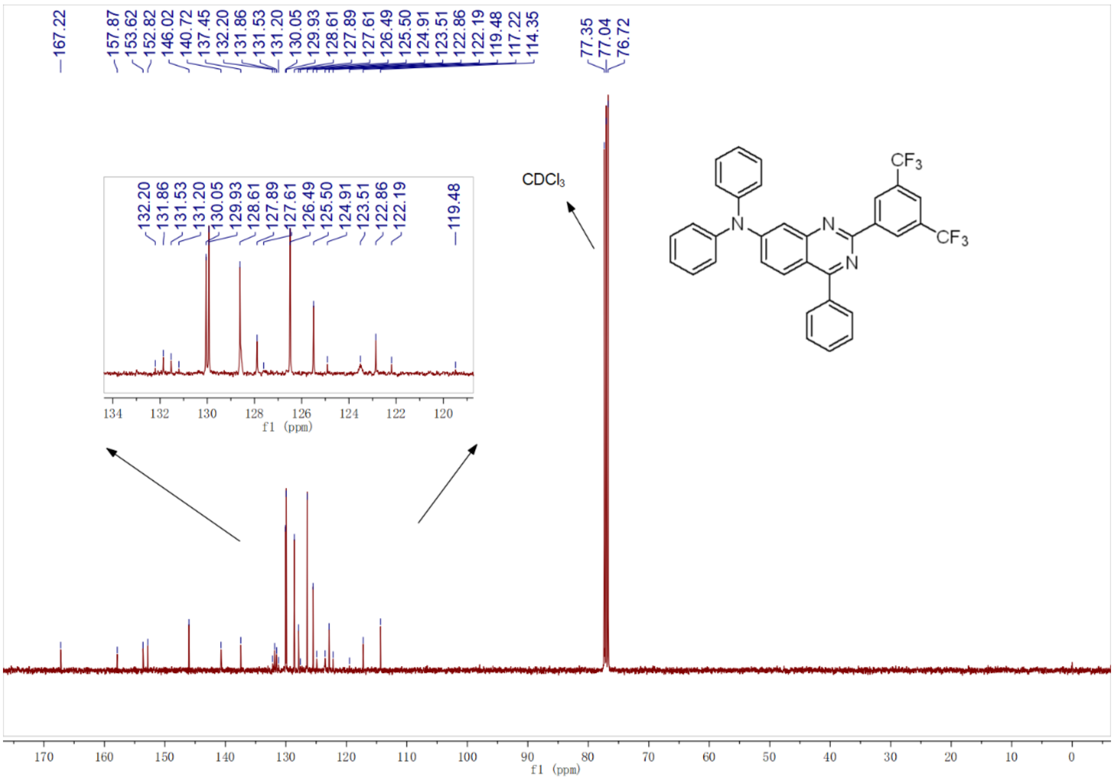

Figure S27 ^{13}C NMR of **7** in CDCl3

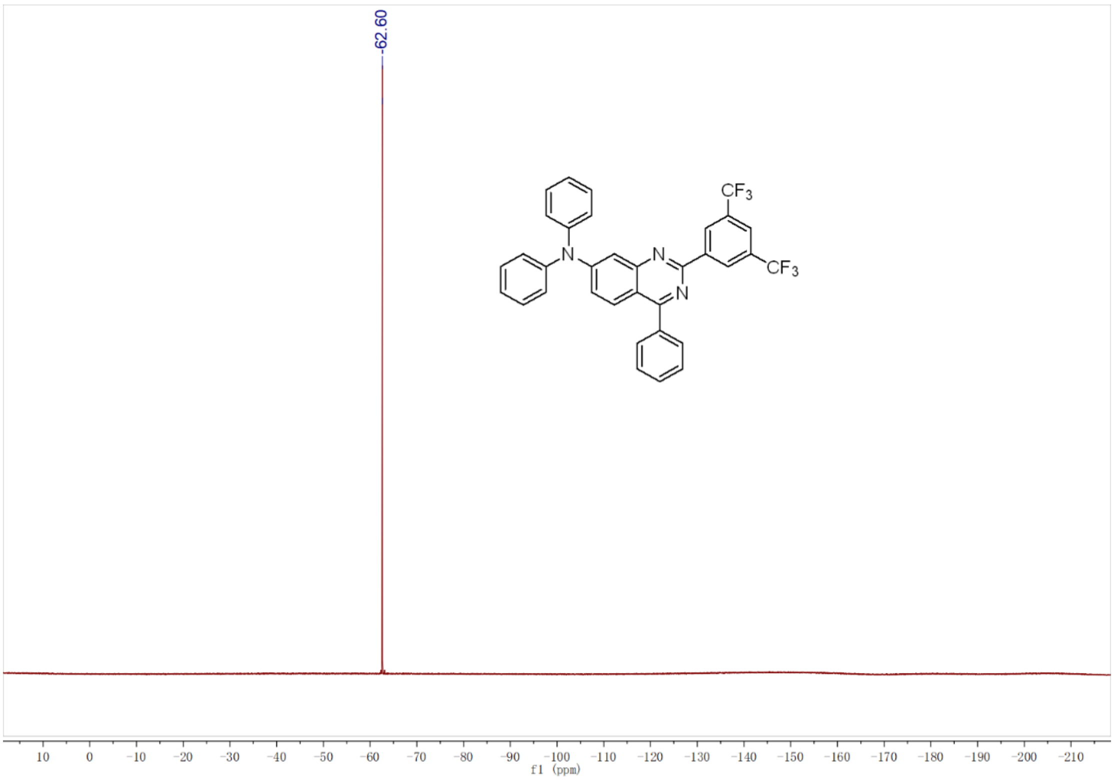

Figure S28 ^{19}F NMR of **7** in $CDCl_3$

Compound 8: DCM:PE=1:2 as eluent, orange solid, yield 86.8%, mp 265-266 °C. ^{1}H NMR (400 MHz, $CDCl_3$) δ 9.09 (s, 2H), 7.94 (s, 1H), 7.89 – 7.81 (m, 3H), 7.62 – 7.55 (m, 3H), 7.27 (d, *J* = 2.4 Hz, 1H), 7.22 (d, *J* = 8.9 Hz, 4H), 7.17 (dd, *J* = 9.3, 2.4 Hz, 1H), 6.95 (d, *J* = 8.9 Hz, 4H), 3.85 (s, 6H).^{13}C NMR (101 MHz, $CDCl_3$) δ 166.9, 157.8, 157.6, 153.7, 153.5, 140.9, 138.7, 137.6, 131.6 (q, *J* = 33.2 Hz), 130.0, 129.9, 128.6, 128.1, 127.8, 123.6 (q, *J* = 272.9 Hz), 123.4, 121.3, 116.4, 115.2, 111.3, 55.56.^{19}F NMR (376 MHz, $CDCl_3$) δ -62.58.HRMS (Maldi-TOF): cacld. for $C_{36}H_{25}F_6N_3O_2$ $[M+H]^+$, 645.1851; found: 645.1858. IR:3067, 2994,1605, 1508, 1484, 1406, 1279, 1245, 1133, 828, 700, 682.

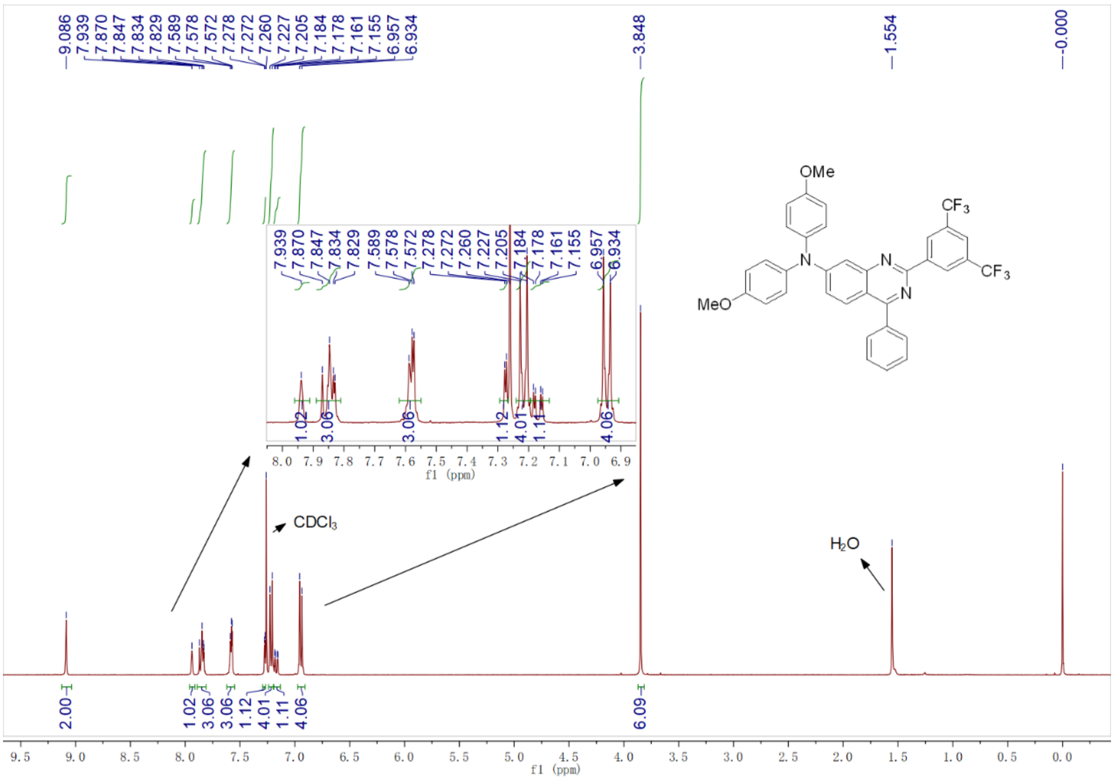

Figure S29 ^{1}H NMR of **8** in $CDCl_3$

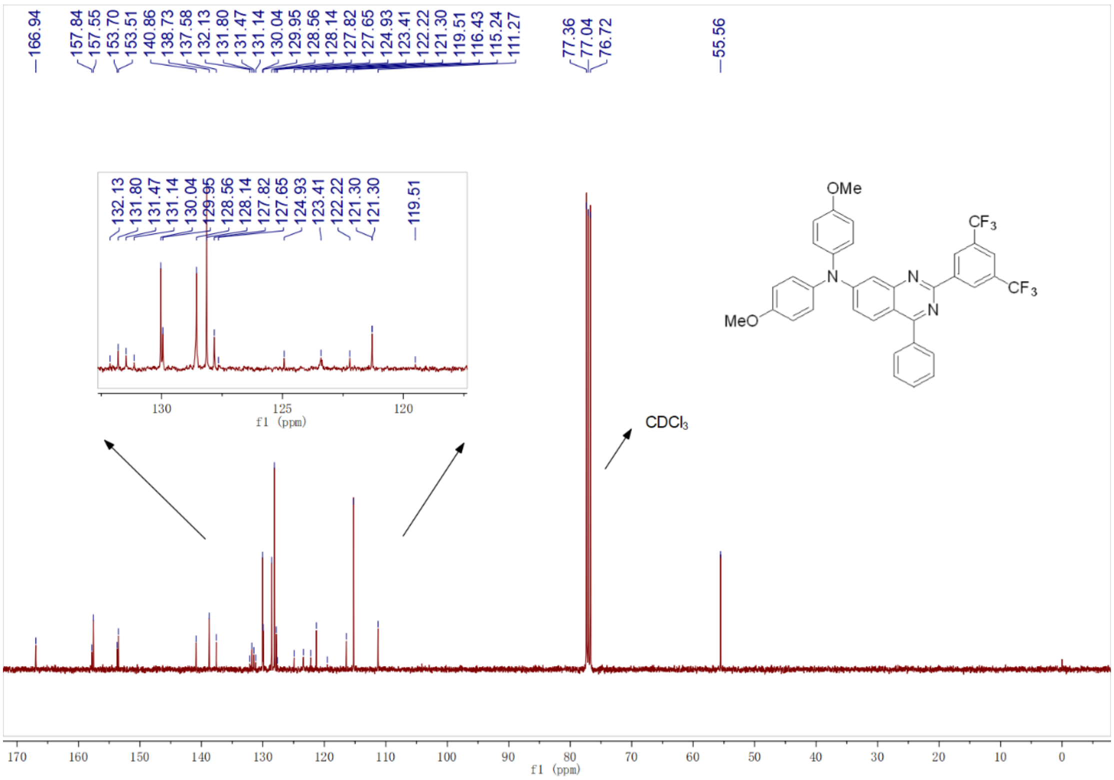

Figure S30 ^{13}C NMR of **8** in $CDCl_3$

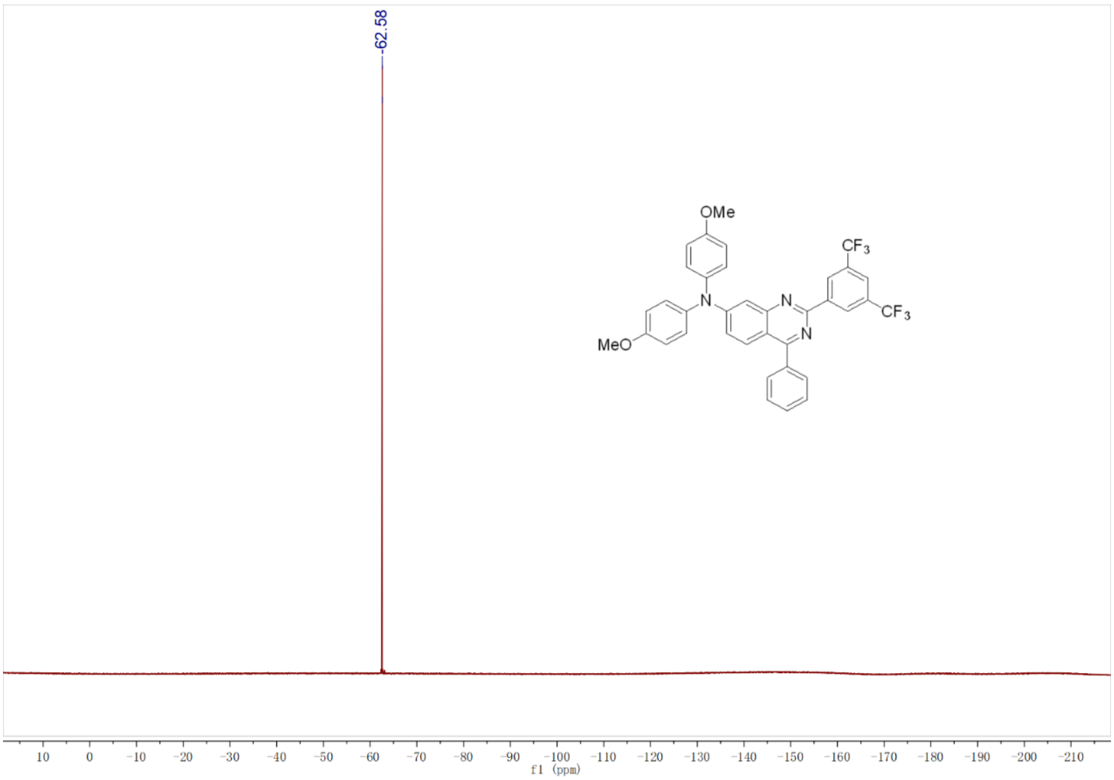

Figure S31 ^{19}F NMR of **8** in $CDCl_3$

Compound 9: DCM:PE=1:4 as eluent, dark orange solid, yield >99%, mp 286-287 °C. ^{1}H NMR (400 MHz, $CDCl_3$) δ 9.20 (s, 2H), 8.39 (d, *J* = 8.9 Hz, 1H), 8.27 (d, *J* = 2.0 Hz, 1H), 8.02 (s, 1H), 7.94 (dd, *J* = 6.6, 2.9 Hz, 2H), 7.71 – 7.65 (m, 3H), 7.61 (dd, *J* = 8.9, 2.0 Hz, 1H), 6.82 – 6.71 (m, 4H), 6.65 (m, 2H), 6.16 (d, *J* = 7.9 Hz, 2H).^{13}C NMR (101 MHz, $CDCl_3$) δ 169.0, 157.9, 153.6, 144.9, 144.5, 140.0, 136.8, 133.2, 132.0 (q, *J* = 33.5 Hz), 130.7, 130.6, 130.5, 130.2, 130.0, 128.9, 128.8, 124.1, 123.5 (q, *J* = 273.1 Hz), 123.4, 122.5, 121.5, 116.0, 114.0.^{19}F NMR (376 MHz, $CDCl_3$) δ -62.67.HRMS (Maldi-TOF): cacld. for $C_{34}H_{19}F_6N_3O[M+H]^+$, 599.1432; found: 599.1431. IR:3080, 2916, 1610, 1488, 1390, 1324, 1280, 1130, 742, 700, 683.

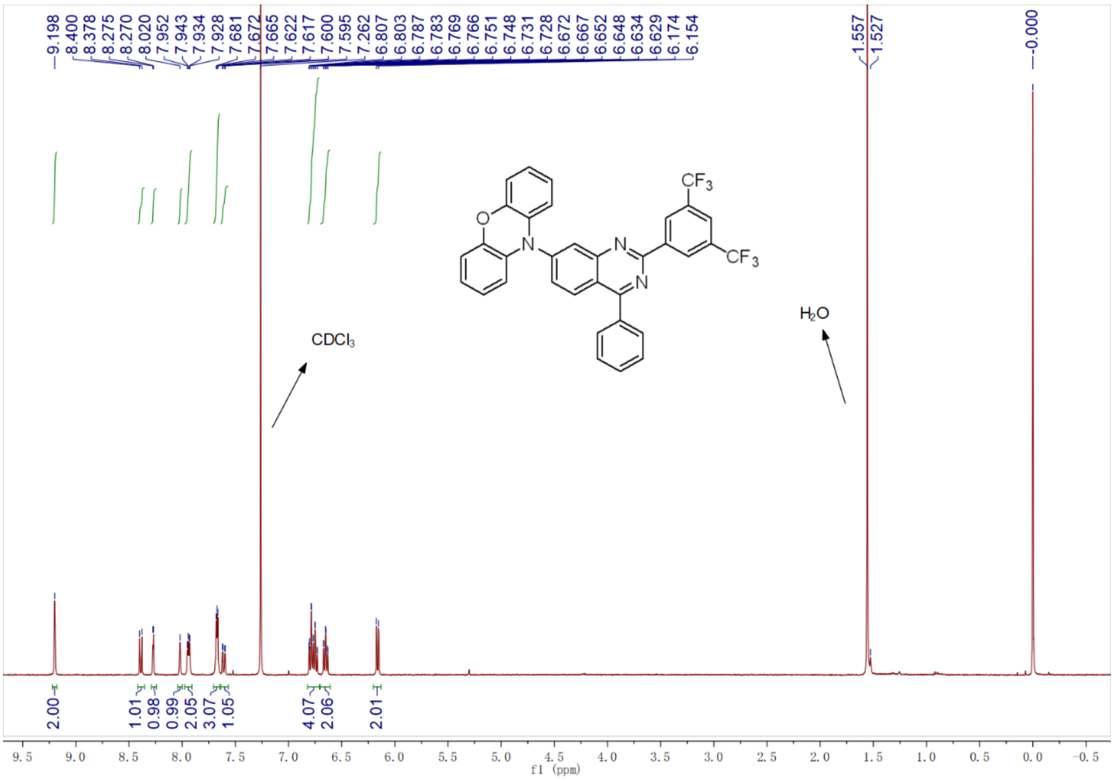

Figure S32 ^{1}H NMR of **9** in $CDCl_3$

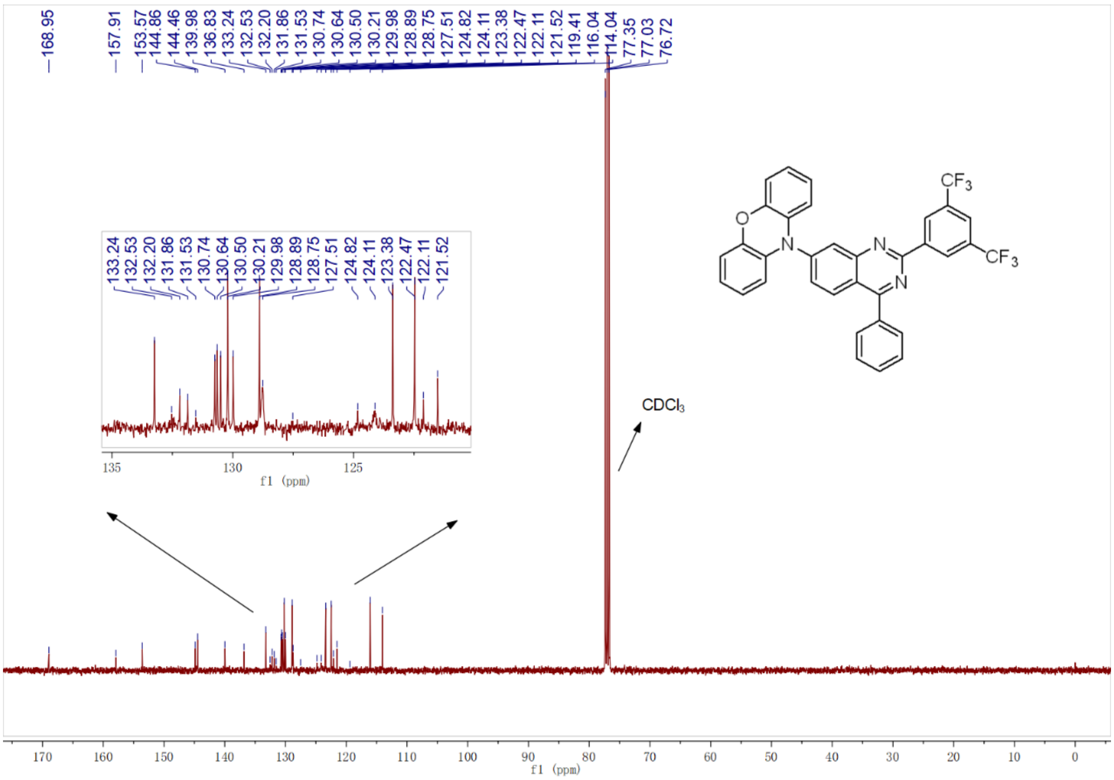

Figure S33 ^{13}C NMR of **9** in $CDCl_3$

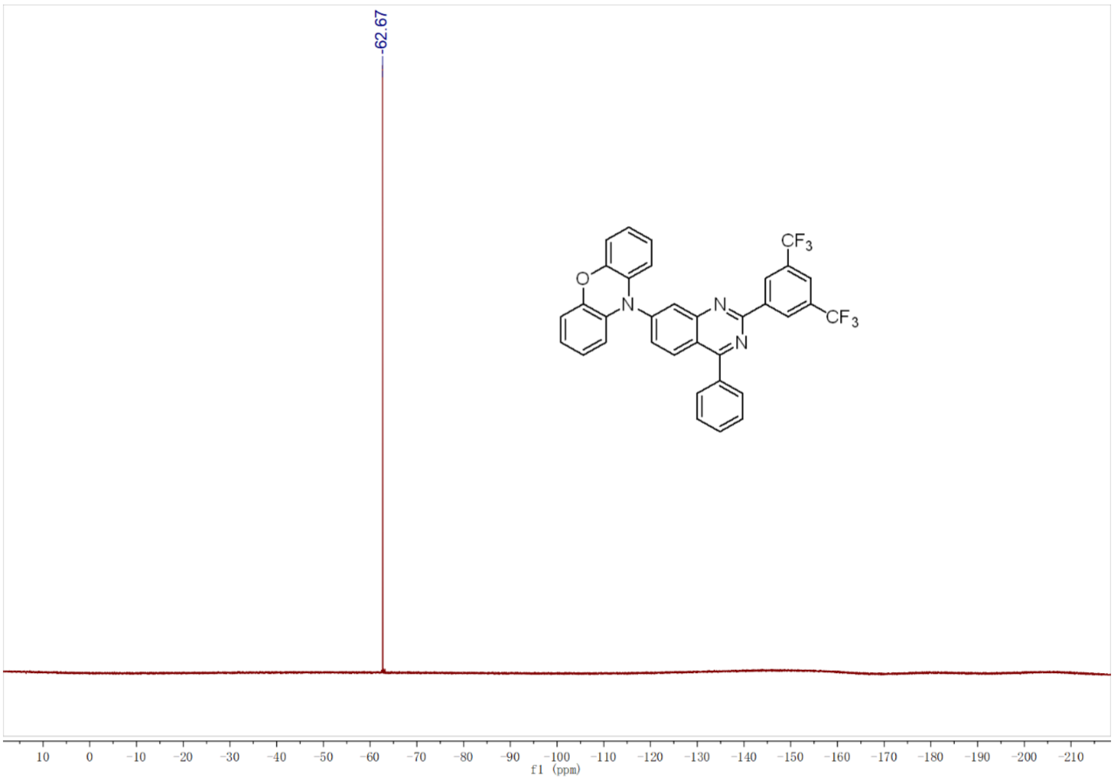

Figure S34 ^{19}F NMR of **9** in $CDCl_3$

Compound 10: DCM:PE=1:1 as eluent, orange red solid, yield 86.6%, mp 248-249°C. ^{1}H NMR (400 MHz, $CDCl_3$) δ 9.08 (s, 2H), 7.93 (s, 1H), 7.87 – 7.78 (m, 3H), 7.57 (dd, *J* = 5.0, 1.8 Hz, 3H), 7.24 (s, 1H), 7.17 (d, *J* = 7.9 Hz, 5H), 6.75 (d, *J* = 8.6 Hz, 4H), 2.99 (s, 12H).^{13}C NMR (101 MHz, $CDCl_3$) δ 166.6, 157.7, 148.7, 141.1, 137.8, 135.1, 131.6 (q, *J* = 33.3 Hz), 130.0, 129.8, 128.5, 127.9, 127.6, 123.6 (q, *J* = 272.8 Hz), 123.3, 121.1, 116.0, 113.6, 109.83, 40.8.^{19}F NMR (376 MHz, $CDCl_3$) δ -62.56. HRMS (Maldi-TOF): cacld. for $C_{38}H_{31}F_6N_5[M+H]^+$, 671.2484; found: 671.2481. IR:3028, 2800, 1616, 1519, 1483, 1325, 1278, 1132, 816, 700, 682.

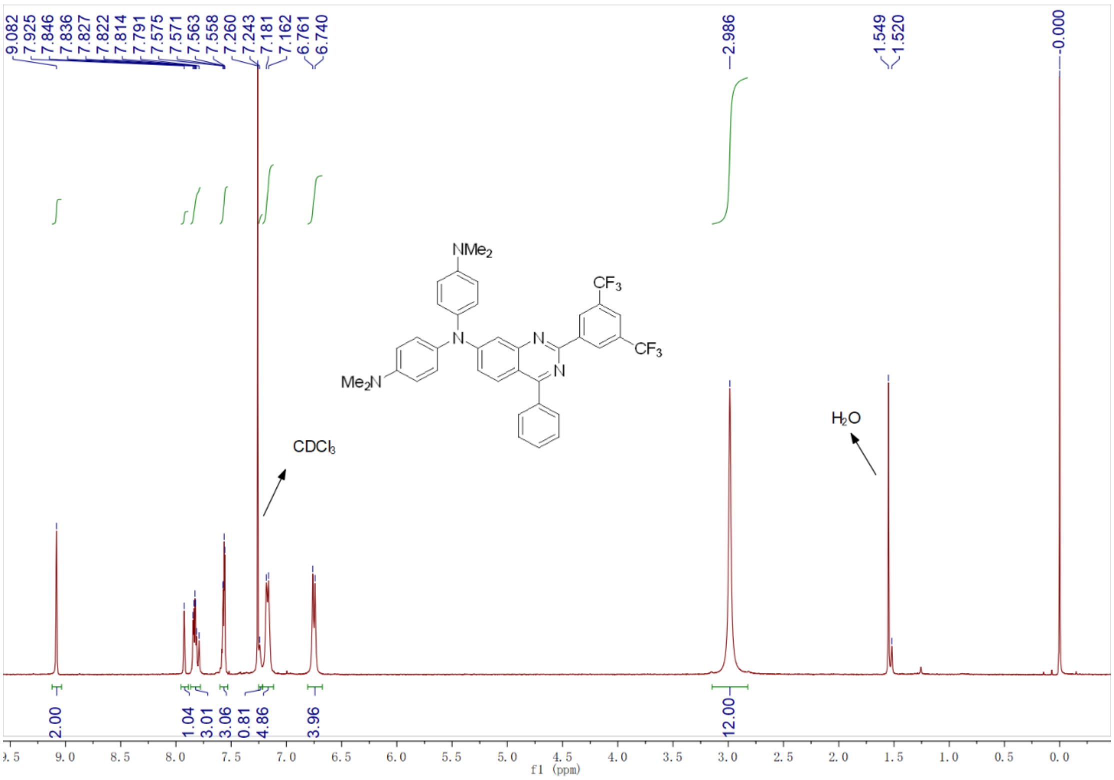

Figure S35 ^{1}H NMR of **10** in $CDCl_3$

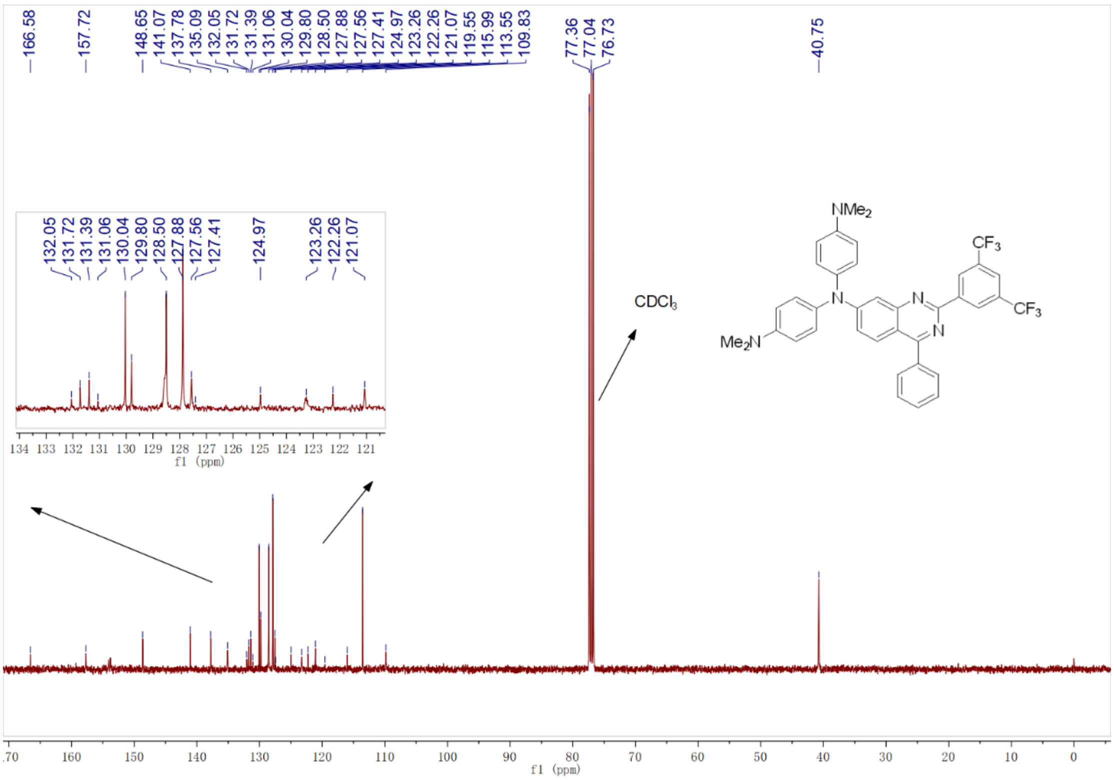

Figure S36 ^{13}C NMR of **10** in $CDCl_3$

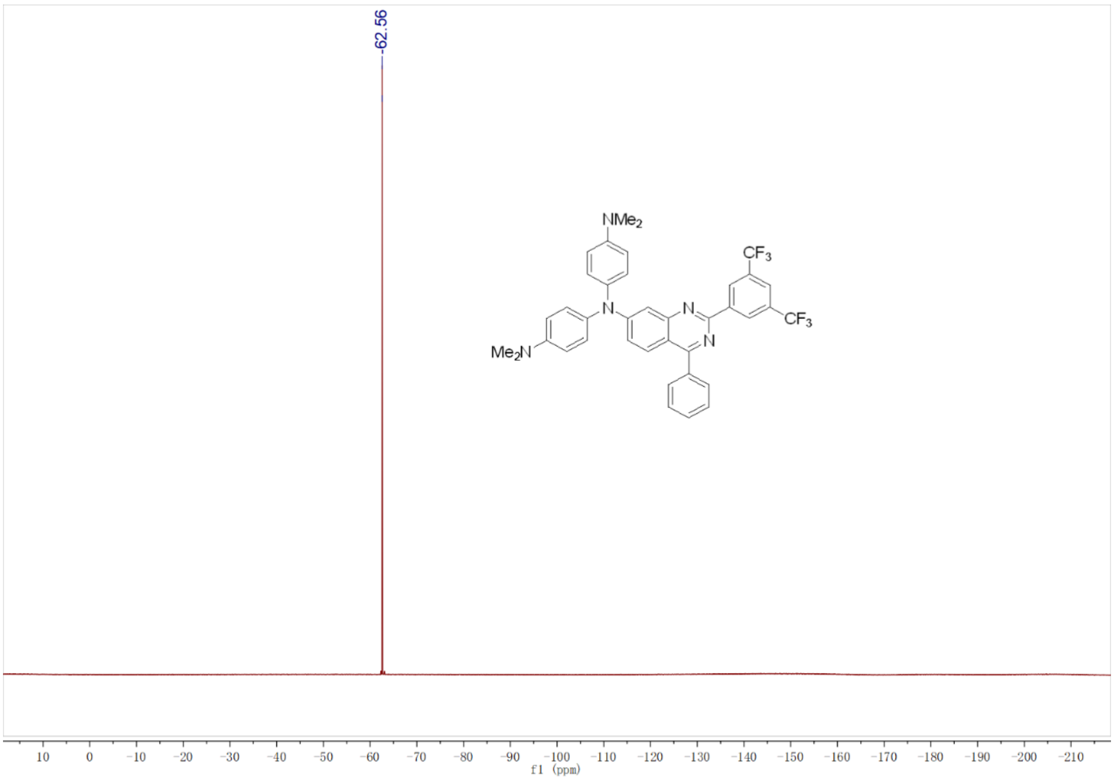

Figure S37 ^{19}F NMR of **10** in $CDCl_3$

Table S1 Single crystal data of compounds**1-4**

**Compunds**	**1**	**2**	**3**	**4**
Formula	$C_{34}H_{19}F_6N_3$	$C_{34}H_{21}F_6N_3$	$C_{36}H_{25}F_6N_3O_2$	$C_{34}H_{19}F_6N_3O$
CCDC	1961660	1961661	1961663	1961672
a [Å]	10.3025	9.459	9.488	10.347
b [Å]	15.7894	12.647	9.778	11.159
c [Å]	15.9352	13.633	16.834	26.443
α [°]	90	92.321	105.688	90
β [°]	92.997	107.307	97.397	93.920
γ [°]	90	92.887	94.639	90
Z	4	2	2	4
V [Å^3]	2588.6	1552.4	1480.1	3072.5
D [g cm^{-3}]	1.497	1.253	1.449	1.480
Space group	P 21/c	P -1	P -1	P 21/c
Hall group	-P 2ybc	-P 1	-P 1	-P 2ybc
Mu [mm^{-1}]	0.119	0.099	0.117	0.283
R (reflections)	0.0494	0.0622	0.0467	0.0541
wR2 (reflections)	0.1422	0.1796	0.1248	0.1463

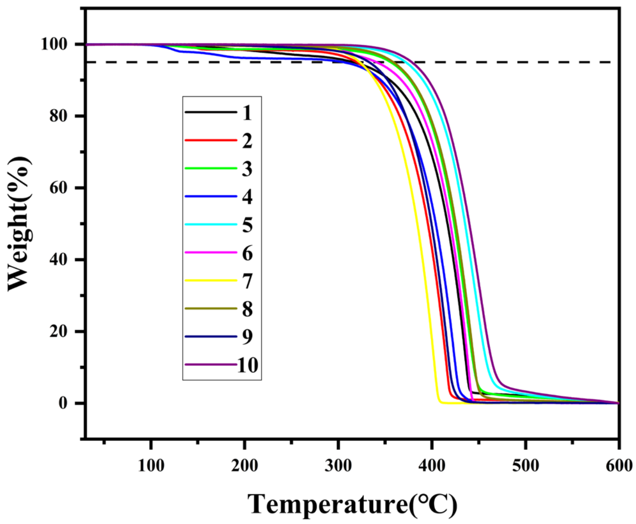

Figure S38 Thermogravimetric Analysis of ten compounds.

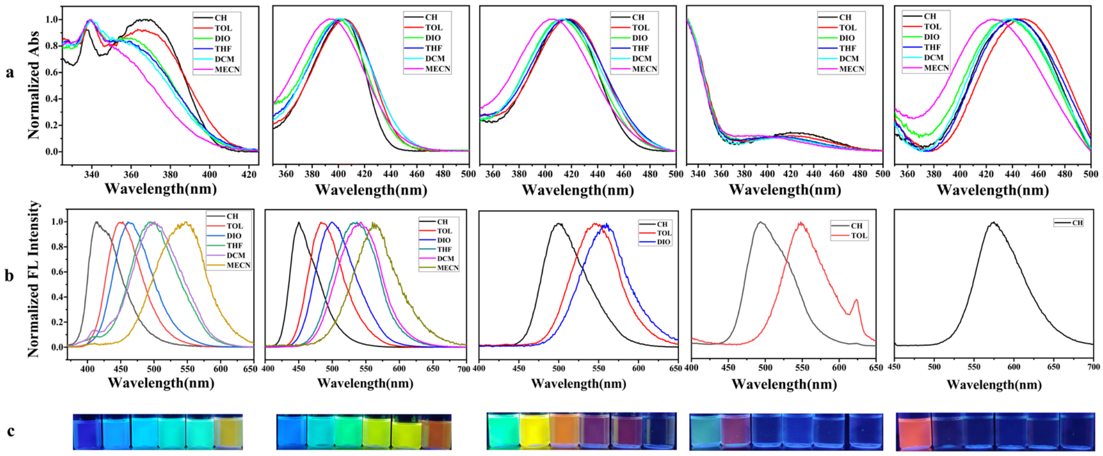

Figure S39 Photophysical properties in different solvent: (a) absorption of **1-5** (b) emission spectra of **1-5** (excited at its corresponding maximum absorption wavelength), (c) photos of **1-5** under UV lamp (365 nm).

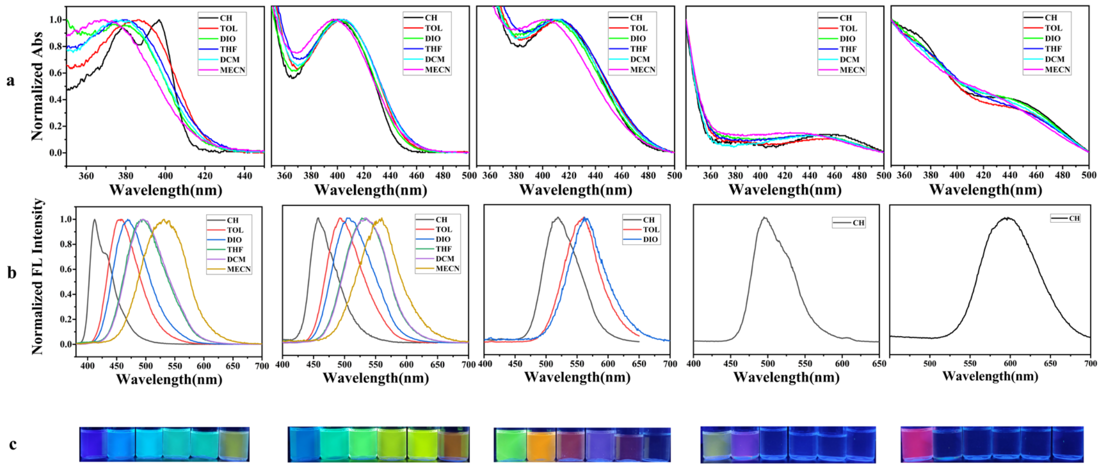

Figure S40 Photophysical properties in different solvent: (a) Absorption of **6-10** (b) emission spectra of **6-10** (excited at its corresponding maximum absorption wavelength), (c) photos of **6-10** under UV lamp (365 nm).

Table S2 Solvent effect on absorption and emission of ten compounds.

		CH	Tol	DIO	THF	DCM	MeCN
**1**	Abs(nm)	338, 369	340, 363	339, 358	339, 354	340, 354	339
	FL(nm)	414	453	461	495	501	548
	Stokes(nm)	45	90	103	141	147	209
**2**	Abs(nm)	403	405	401	402	401	393
	FL(nm)	450	483	499	537	543	562
	Stokes(nm)	47	78	98	135	142	169
**3**	Abs(nm)	415	419	413	416	413	405
	FL(nm)	500	545	560			
	Stokes(nm)	85	126	147			
**4**	Abs(nm)	420					
	FL(nm)	514	568				
	Stokes(nm)	94					
**5**	Abs(nm)	444	448	436	442	438	425
	FL(nm)	575					
	Stokes(nm)	131					
**6**	Abs(nm)	378, 397	386	381	380	375	369
	FL(nm)	412	456	469	495	495	531
	Stokes(nm)	15	70	88	115	120	162
**7**	Abs(nm)	400	405	401	402	403	399
	FL(nm)	458	493	504	528	534	560
	Stokes(nm)	58	88	103	126	131	161
**8**	Abs(nm)	411	410	407	411	410	400
	FL(nm)	519	561	562			
	Stokes(nm)	108	151	155			
**9**	Abs(nm)	456					
	FL(nm)	533					
	Stokes(nm)	77					
**10**	Abs(nm)	436					
	FL(nm)	597					
	Stokes(nm)	161					

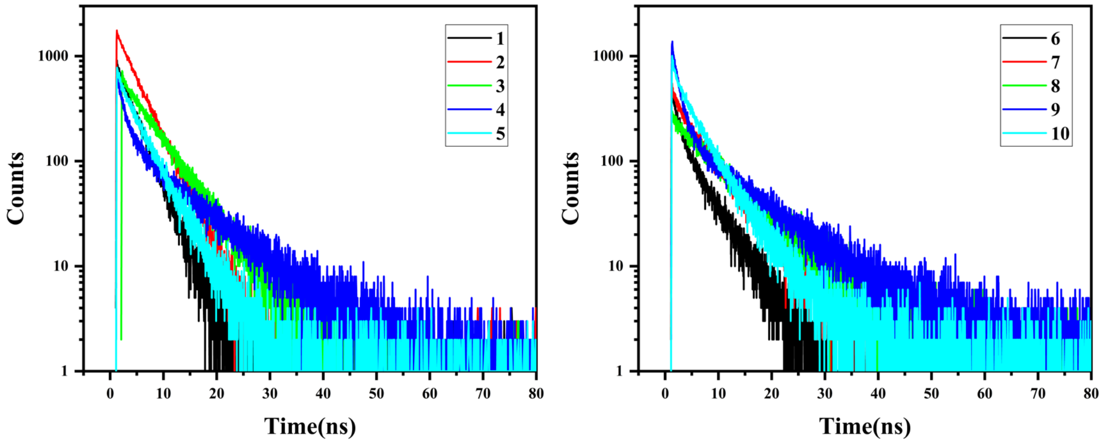

Figure S41 Fluorescence decay traces of ten compounds in cyclohexane (compounds 5 and 10) or toluene (other compounds) at 2 x 10^{-5} M.

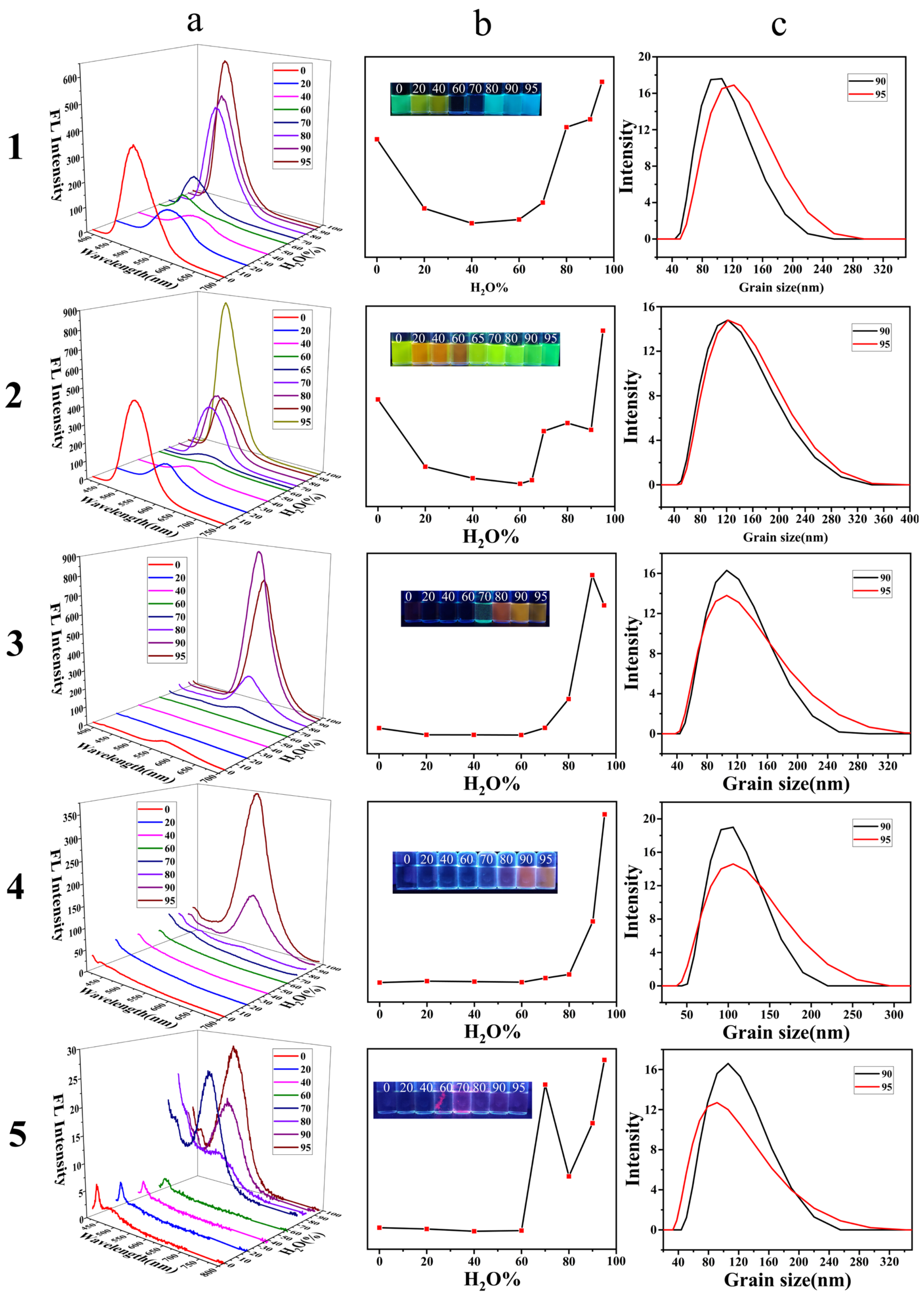

Figure S42 (a) 3D Emission spectrum in aqueous THF solutions, (b) the relative intensity and photos and (c) DLS (fw = 90%, 95%) of compounds **1-5**.

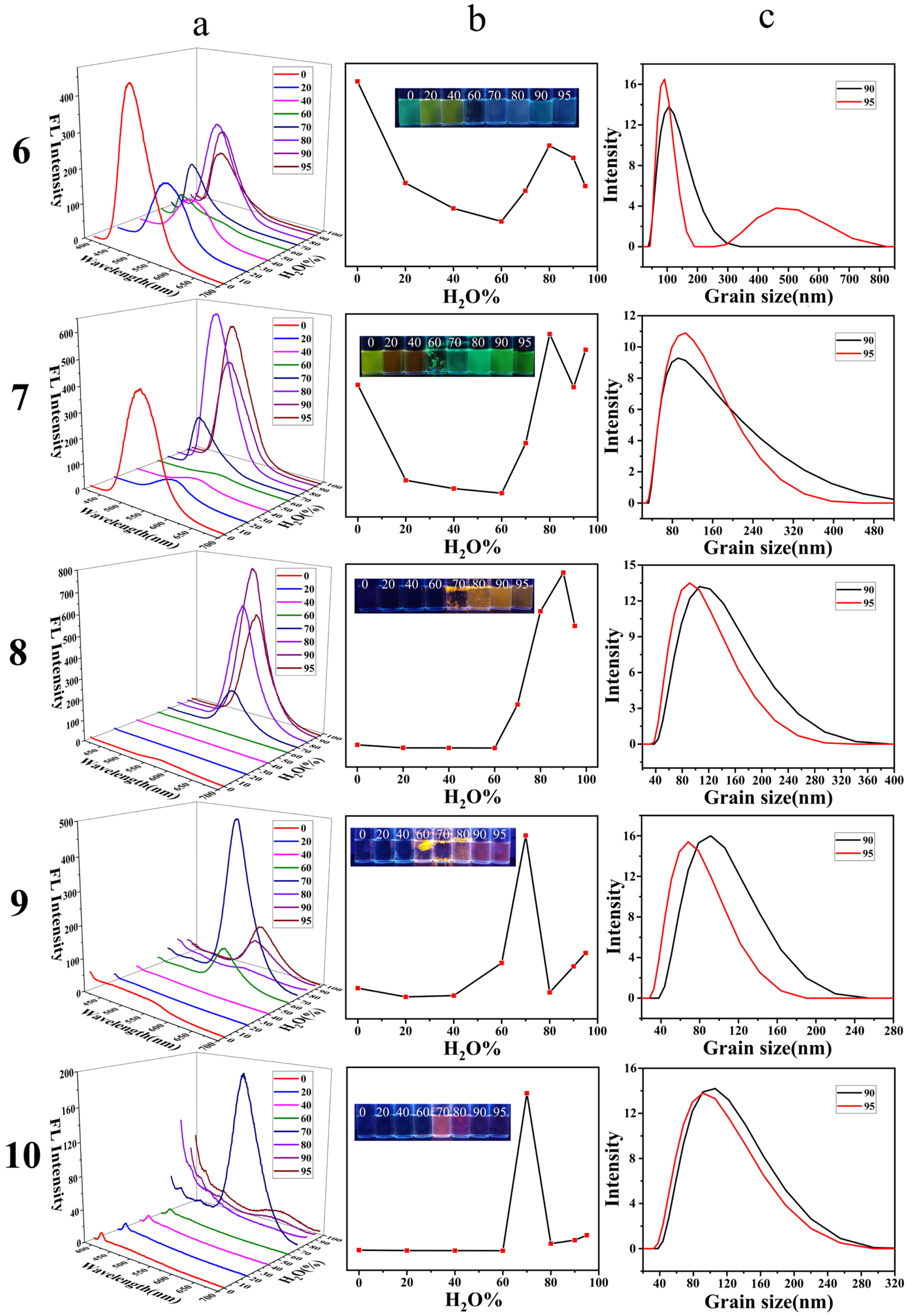

Figure S43 (a) 3D Emission spectrum in aqueous THF solutions, (b) the relative intensity and photos and(c) DLS (fw = 90%, 95%) of compounds **6-10**.

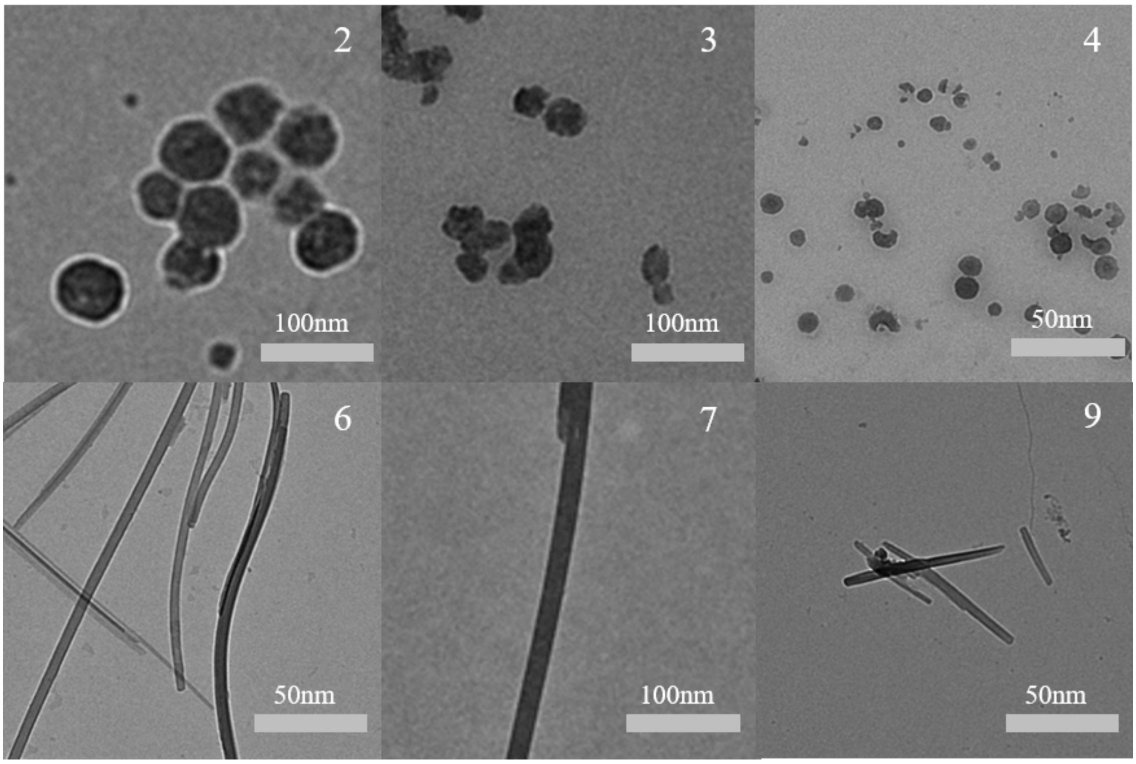

Figure S44 TEM image in aqueous THF solutions of **2** (fw = 95%),**3** (fw = 95%),**4** (fw = 90%),**6** (fw = 80%),**7** (fw = 80%), **9** (fw = 95%).

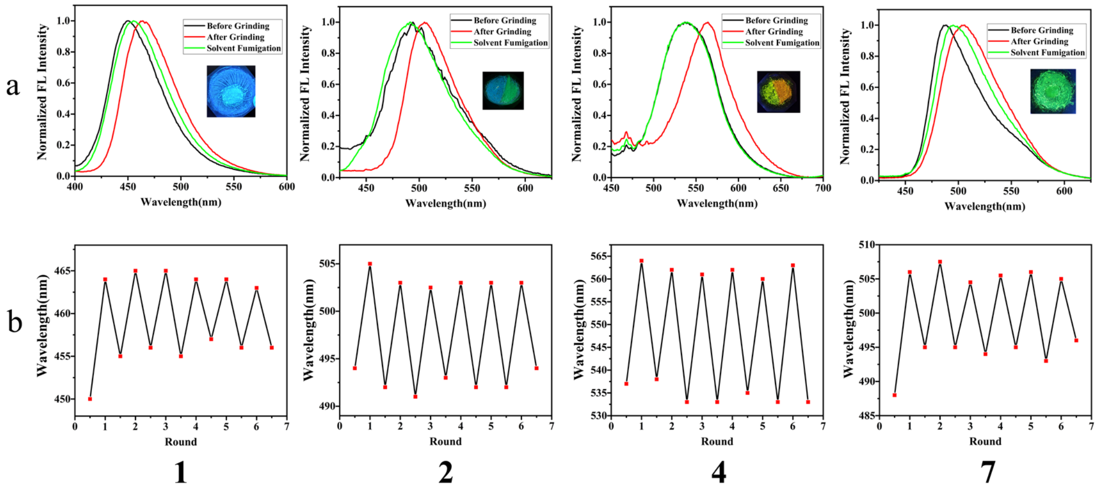

Figure S45 Mechanochromic property of compounds **1, 2, 4** and **7**; a) normalized emission spectra; b) emission cycles.

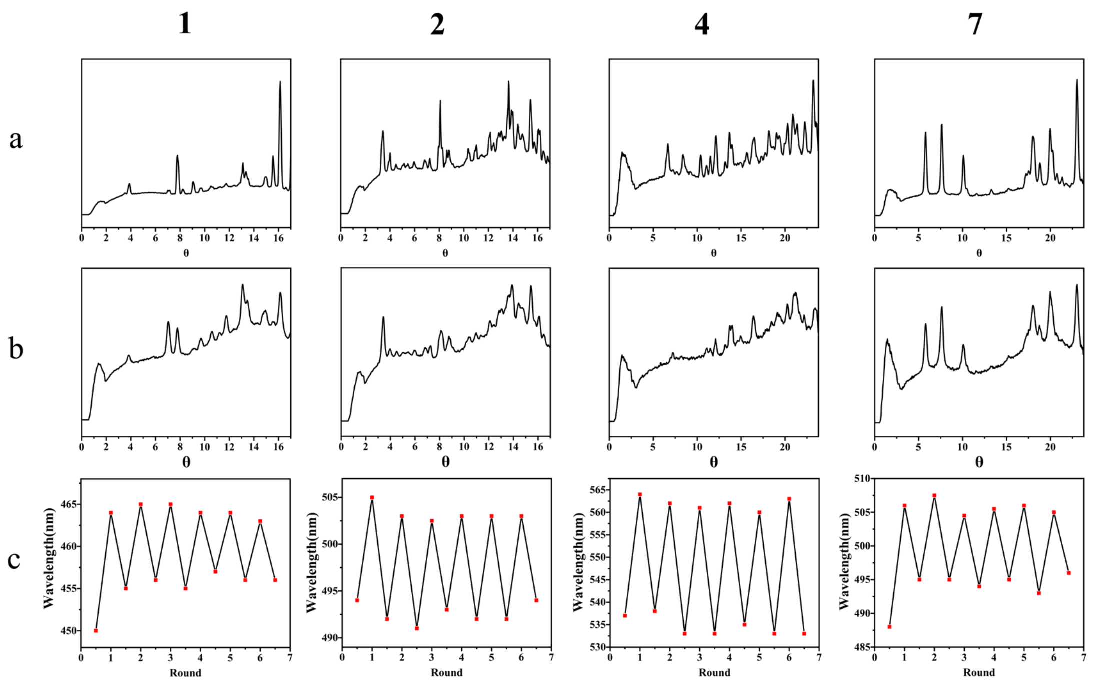

Figure S46 SAXS analysis in the cycle of pristine (a) and ground (b), repeatable cycles, monitored by emission spectra (c) emission cycles.

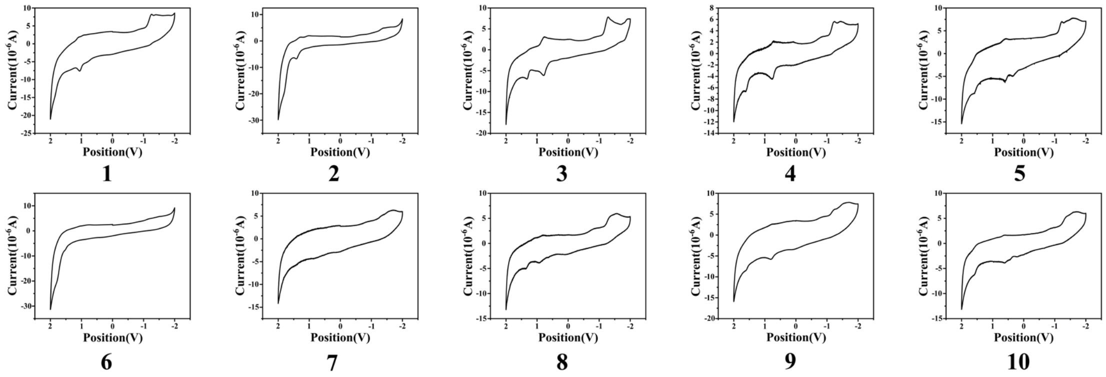

Figure S47 Cyclic voltammograms of ten compounds.

Table S3 HOMO/LUMO levels calculated from theoretical calculation and CV as well as UV measurements.

Compound	HOMO		LUMO		E_g		
	CV[a] (eV)	Cald[b] (eV)	CV[c] (eV)	Cald[b] (eV)	CV (eV)	Cald (eV)	UV[d] (eV)
**1**	-5.35	-5.15	-3.30	-2.06	2.05	3.09	3.00
**2**	-5.79	-5.45	-3.16	-2.22	2.63	3.23	2.77
**3**	-5.18	-4.88	-3.28	-1.98	1.90	2.90	2.62
**4**	-5.15	-4.72	-3.29	-2.27	1.86	2.45	2.63
**5**	-4.73	-4.46	-3.30	-1.9	1.43	2.56	2.48
**6**	\	-5.55	\	-2.24	\	3.31	2.93
**7**	\	-5.35	-3.25	-1.98	\	3.37	2.73
**8**	-5.38	-5.05	-3.23	-1.89	2.15	3.16	2.56
**9**	-5.16	-4.80	-3.31	-2.3	1.85	2.50	2.49
**10**	-4.85	-4.57	-3.18	-1.78	1.67	2.79	2.48

[a] Potentials vs reference electrode SCE, working electrode glassy carbon, auxiliary electrode Pt, 0.1 M $Bu_4N^+PF6^-$DCM, scan rate 100 mV/s, HOMO = E_{ref}-E_{ox}. [b] Obtained from DFT using the B3LYP functional and the 6-31G(d) basis set. [c] LUMO= E_{ref}-E_{red}. [d]E_g= 1240 / λ ($UV_{onset\ in\ CH}$).

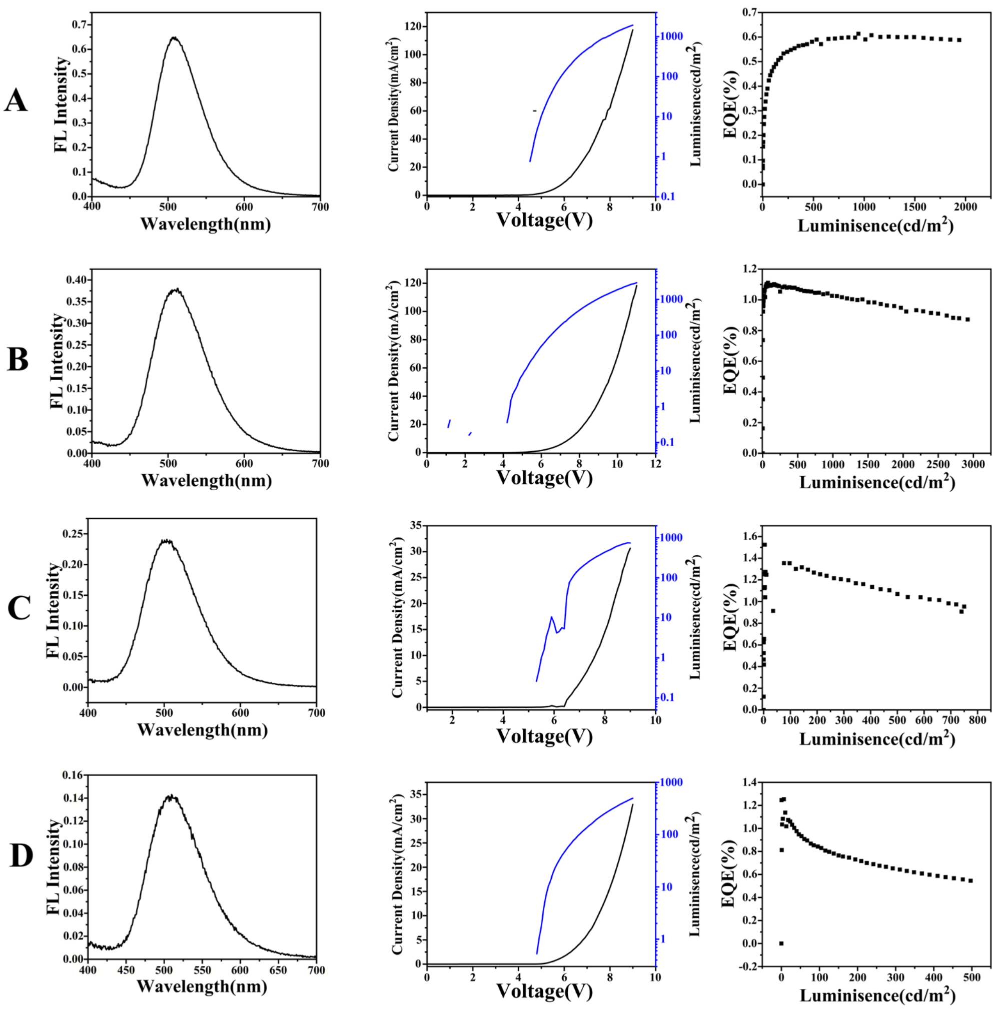

Figure S48 C-I-V curves of devices A, B, C and D.

Table S4 Device performance

Device	Emitter	$V_{turn-on}$(V)[a]	CIE_{1931} (x,y)	λ_{EL}(nm)	EQE(%)[b]	CE(cd/A)[b]	PE(lm/W)[b]
A 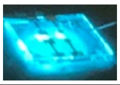	**2**	4.5	(0.21,0.54)	509	0.47	1.35	0.72
B 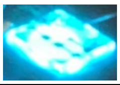	**2**:PVK=1:4	4.2	(0.23,0.51)	513	1.09	3.23	1.56
C 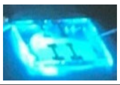	**2**:PVK=1:8	5.3	(0.20,0.47)	497	1.35	3.72	1.74
D 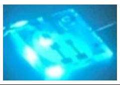	**7**:PVK=1:4	4.8	(0.23,0.50)	510	0.83	2.45	1.16

[a] Applied voltage at the luminance of 1 cd/m^2.

[b] Value at 100 cd/m^2.

Reference

1. K. Hu, Q. Zhen, J. Gong, T. Cheng, L. Qi, Y. Shao and J. Chen, *Org Lett*, 2018, **20**, 3083-3087.
